# Substituent Effects on the Stability and Antioxidant Activity of Spirodiazaselenuranes

**DOI:** 10.3390/molecules200712959

**Published:** 2015-07-17

**Authors:** Devappa S. Lamani, Debasish Bhowmick, Govindasamy Mugesh

**Affiliations:** Department of Inorganic and Physical Chemistry, Indian Institute of Science, Bangalore-560012, India; E-Mails: kattimani.devaraj67@gmail.com (D.S.L.); debasish@ipc.iisc.ernet.in (D.B.)

**Keywords:** spirodiazaselenuranes, antioxidant, glutathione peroxidase, peroxynitrite

## Abstract

Spirodiazaselenuranes are structurally interesting compounds and the stability of these compounds depends highly on the nature of the substituents attached to the nitrogen atoms. Aromatic substituents are known to play important roles in stabilizing the Se-N bonds in spiro compounds. In this study, several spirodiazaselenuranes are synthesized by introducing benzylic and aliphatic substituents to understand their effect on the stability of the Se-N bonds and the antioxidant activity. Replacement of phenyl substituent by benzyl/alkyl groups significantly reduces the stability of the spirodiazaselenuranes and slows down the oxidative cyclization process. The selenium centre in the spiro compounds undergoes further oxidation to produce the corresponding selenurane oxides, which are stable at room temperature. Comparison of the glutathione peroxidase (GPx) mimetic activity of the compounds showed that the diaryl selenides having heterocyclic rings are significantly more active due to the facile oxidation of the selenium centre. However, the activity is reduced significantly for compounds having aliphatic substituents. In addition to GPx activity, the compounds also inhibit peroxynitrite-mediated nitration and oxidation reaction of protein and small molecules, respectively. The experimental observations suggest that the antioxidant activity is increased considerably upon substitution of the aromatic group with the benzylic/aliphatic substituents on the nitrogen atoms.

## 1. Introduction

**Figure 1:**
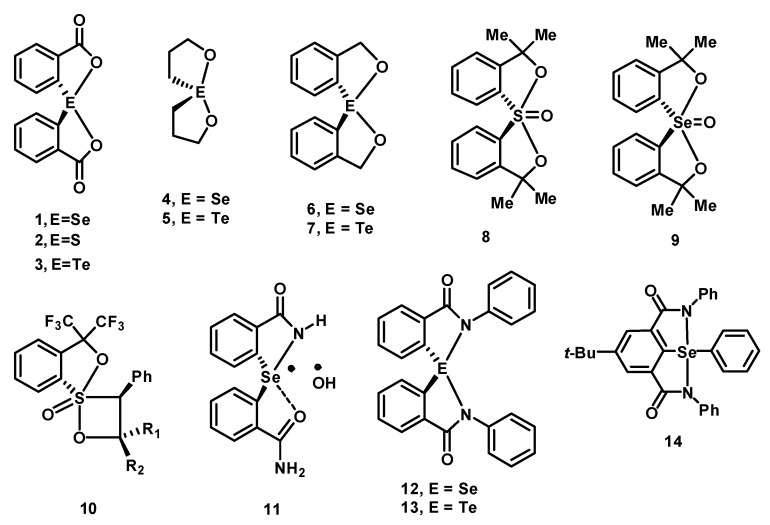
Some representative examples of stable spirochalcogenuranes.

Spirodiazaselenuranes have attracted considerable attention because of their interesting structural and stereochemical properties [1,2,3,4,5]. Spirodiazaselenuranes are structurally similar to spirodioxyselenurane **1**, which was reported by Lesser and Weiss in 1914 [5]. The corresponding sulphur and tellurium analogues **2**–**3** were also reported in the literature (Figure 1) [6,7]. Subsequently, a series of spirodioxyselenuranes with different stereochemistry and ring size has been reported [8,9,10,11]. In 2004, Back and co-workers showed for the first time that spirodioxyselenurane **4** and its tellurium analogue **5** exhibit very good antioxidant activity by mimicking the glutathione peroxidase (GPx) enzyme [12], which protects the organism from oxidative damage by catalyzing the reduction of peroxides using thiol as the cofactor [13,14]. However, the corresponding aromatic derivatives **6**–**7** were found to be much less active than the aliphatic compounds **4**–**5** [15,16]. Several sulfurane oxides and selenurane oxides such as **8**–**10**, in which the chalcogen atoms are in the higher oxidation state, were also synthesized and isolated (Figure 1) [17,18,19]. Although several example of spirodioxyselenuranes are available in the literature, examples of stable spirodiazaselenuranes are very rare. Spirodiazaselenuranes are highly unstable and an earlier attempt to synthesize a spirodiazaselenurane produces the corresponding azaselenonium hydroxide (**11**) containing only one Se-N bond [20]. Recently, our group reported a stable spirodiazaselenurane **12** and its tellurium analogue **13** obtained by substituting the hydrogen of the amide moiety with a phenyl substituent [21]. The geometry around the selenium or tellurium atom is trigonal bipyramidal, where the two apical positions are occupied by the nitrogen atoms and one of the equatorial positions is occupied by the lone electron pair of the central atom [5,8]. It is proposed that the phenyl substituent on the nitrogen atom is important, as it facilitates the formation of the diazaselenuranes by increasing the stability of the Se-N bonds. Furthermore, our previous study by substituting the hydrogen atom on the phenyl group with different electron donating and electron withdrawing atoms or groups showed that the electron donating substituents generally enhance the stability of the Se-N bonds and their antioxidant activity [22]. Very recently, Singh and co-workers have synthesized and characterized a new pincer type bicyclic diazaselenurane **14** in which the two amide groups are present in the same phenyl ring forming the bicycles [23].

Earlier reports show that oxidation of selenides by H_2_O_2_ produces the corresponding selenoxides, which undergo a rapid cyclization via a hydroxyselenurane intermediate to produce the spirodiazaselenuranes [21,22]. The aromatic ring on the nitrogen plays an important role in the cyclization by stabilizing the Se-N bonds in the spirodiazaselenurane. In the present study, we have substituted the phenyl/substituted phenyl group with benzylic (**29**), pyridylmethyl (**30**–**31**), heterocyclic (**32**–**33**) and aliphatic side chains (**34**–**35**) at the nitrogen atoms to understand their influence on the cyclization and stability of the spirodizaselenuranes. Although selenurane oxides with two oxygen atoms at the axial positions are known, such compounds with two nitrogen atoms bonded to selenium are still unknown. In this regard, the stable selenurane oxide reported in this paper is novel. We also report on the GPx-like activity and peroxynitrite scavenging activity of new compounds.

## 2. Results and Discussion

### 2.1. Synthesis of Spirodiazaselenuranes

**Scheme 1:**
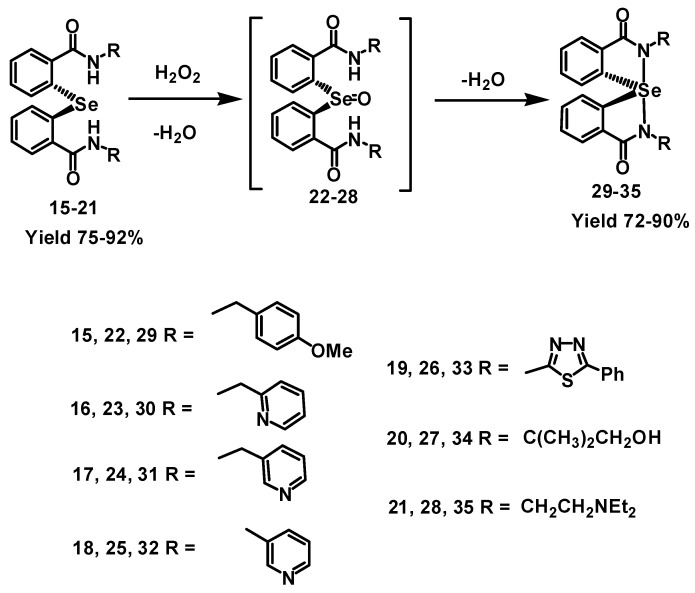
The diaryl selenides **15**–**21** were synthesized from the 2,2′-selenodibenzoyl chloride by reaction with the respective amines. Compounds **15**–**21** convert to the corresponding spirodiazaselenuranes **29**–**35** in the presence of H_2_O_2_ via selenoxide intermediates **22**–**28**.

2,2′-Selenodibenzoyl chloride, which is the key precursor for the selenides used in this study, was synthesized by the reaction of disodium diselenide and 2-iodobenzoic acid in the presence of copper powder with some slight modifications of the reported procedure [22,24], followed by the reaction with thionyl chloride. Treatment of diaryl selenenyl chloride with various substituted amines produce the selenides **15**–**21** (Scheme 1), which are oxidized to the corresponding selenoxide intermediates **22**–**28** in the presence of H_2_O_2_ or *t*-BuOOH. These selenoxides undergo a spontaneous or base-promoted cyclization with the elimination of a water molecule to generate the corresponding spirodiazaselenuranes **29**–**35** in good yields (Scheme 1). All the spirodiazaselenuranes were characterized by ^1^H-, ^13^C- and ^77^Se-NMR spectroscopy and mass spectrometric analysis.

**Figure 2:**
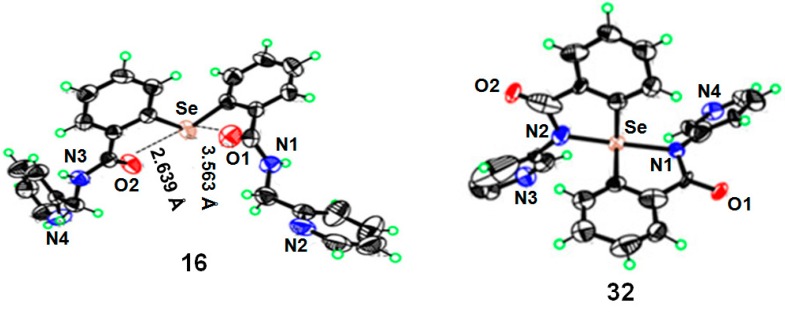
Single crystal X-ray structures of compounds **16** and **32** with atom labeling scheme at 50% probability.

The diaryl selenide **16** and the spirodiazaselenurane **32** were also characterized by the single crystal X-ray diffraction method. The compound **16** is crystallized in the P 21/c space group. The geometry around the selenium atom is V-shaped, with a C1-Se-C14 angle of 102.37° (Figure 2). As two oxygen atoms of the two amide moiety are located near the selenium centre, there is a possibility of non-bonded Se···O interactions as we observe for several amide-based diaryl diselenides [25]. The Se···O1 and Se···O2 distances are 3.563 Å and 2.639 Å, respectively. Although the selenide structure appears to be symmetrical, the Se···O distances are different. The Se···O2 (2.639 Å) distance is much shorter than the sum of the van der Waals radii of selenium and oxygen atoms (3.42 Å). Therefore, one of the two oxygen atoms interacts strongly with the selenium centre and other one weakly interacts with the selenium. Other bond lengths and angles in diaryl selenide **16** are in the normal range observed for the related compounds. The non-covalent Se···O interaction modulates the reactivity of the diaryl selenides toward peroxides and also influences the cyclization process in the selenoxide intermediate. The crystal structure of compound **32** indicates that selenium centre adopts a distorted trigonal bipyramidal (TBP) geometry with the two nitrogen atoms at the apical positions and the two carbon atoms of the aromatic rings and the lone pair on the selenium occupies the equatorial positions (Figure 2) as we have observed previously for other spirodiazaselenurane crystal structures [21].

### 2.2. Mechanism of Cyclization

**Scheme 2:**
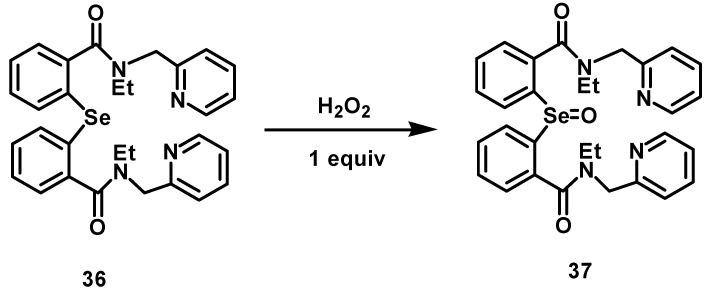
Reaction of compound **36** with H_2_O_2_ to produce selenoxide **37**.

Detailed mechanistic studies for the structural characterization of the spirodiazaselenuranes were carried out using ^77^Se-NMR spectroscopy. It is shown earlier that the cyclization process is very rapid at room temperature (25 °C) in the presence of aromatic substituents on the nitrogen atoms. Therefore, all the reactions were carried out at lower temperature (−25 °C) to isolate the intermediates involved in the process [21,22]. In the present study, it is observed that the diaryl selenides **15**–**21** react with H_2_O_2_ or ONOO^−^ to produce the corresponding selenoxides **22**–**28**, which could be isolated and characterized at room temperature. This indicates that the rate of cyclization is much slower for benzylic and aliphatic substituted selenoxides **22**–**28**. Reaction of the selenide **16** with an equimolar amount of H_2_O_2_ or ONOO^−^ in dichloromethane (CH_2_Cl_2_) for 30 min at room temperature afforded two sharp peaks at 559 ppm and 875 ppm in the ^77^Se-NMR (Figure S14, SI). The peak at 559 ppm is for the spirodiazaselenurane **30**, whereas the other peak at 875 ppm corresponds to the selenoxide **23**. When the reaction mixture was kept for additional 6 h, the peaks corresponding to the diaryl selenide **16** and the selenoxide **23** were completely disappeared with a new peak appearing the spiro compound **30**. However, to confirm the formation of the selenoxide **23**, compound **36** was synthesized from the selenide **16** by substituting the amide hydrogens with ethyl moieties. Upon treatment of one equivalent of H_2_O_2_ with compound **36**, a sharp peak was appeared at 863 ppm in the ^77^Se-NMR (Figure S15, SI). This peak corresponds to the stable selenoxide **37** (Scheme 2), which cannot undergo any cyclization due to the absence of free N-H moiety. The almost similar chemical shift values in the ^77^Se-NMR confirms the formation of stable selenoxide intermediate **23** at room temperature. Therefore, it is clearly noticed that the replacement of the aromatic rings with benzylic (**29**), pyridylmethyl (**30**–**31**), heterocyclic (**32**–**33**) and aliphatic side chains (**34**–**35**) considerably decreases the stability of the Se-N bonds in the spirodiazaselenuranes and thus slows down the cyclization process.

**Scheme 3:**
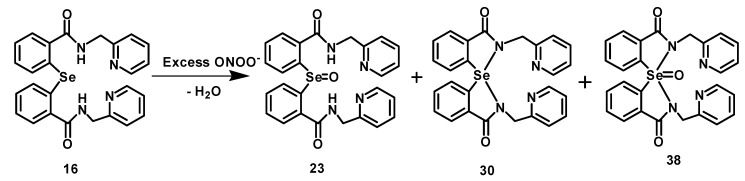
Reaction of **16** with excess of ONOO^−^ produce the spiro compound **30** along with the selenoxide **23** and the selenurane oxide **38**.

Interestingly, when the selenide **16** was treated with excess of peroxynitrite (ONOO^−^), a new peak appeared at 695 ppm in the ^77^Se-NMR spectrum, along with two other peaks for the selenoxide **23** and spiro compound **30** (Figure S16, SI). This new peak corresponds to the selenurane oxide **38**, which is formed after overoxidation of the selenium centre in the spirodiazaselenurane **30** (Scheme 3). Compound **38** was purified by column chromatography and fully characterized by ^1^H-, ^13^C-, ^77^Se-NMR and mass spectral studies (Figures S24–S27, SI). Sulfurane oxide and selenurane oxide with apical oxygen atoms are already known in the literature [17,18,19]. However, compound **38** is the first example of a stable selenurane oxide, which contains two nitrogen atoms at the axial positions. In contrast, the selenium centre undergoes very slow overoxidation in the presence of excess H_2_O_2_ as the oxidizing agent.

### 2.3. Glutathione Peroxidase (GPx)-Like Activity

Spirodioxyselenurane **4** exhibits good antioxidant activity by mimicking glutathione peroxidase enzyme and the activity was found to be higher than that of ebselen (2-phenyl-1,2-benzisoselenazol-3(2*H*)-one) [12]. Similarly, our previous study also showed that some of the spirodiazaselenuranes containing electron donating substituents on the aromatic rings exhibit moderate catalytic activities for the reduction of peroxides [22]. Therefore, the GPx-like catalytic activities of all the selenides **15**–**21** and the spirodiazaselenuranes **29**–**35** were studied using H_2_O_2_ as the substrate and glutathione (GSH) as the co-substrate to understand the effect of different benzylic, heterocyclic and aliphatic substituents on the antioxidant activity. Initial rates (v_0_) for the reduction of peroxide were determined in the presence of different catalysts at 80 µM concentration. Ebselen and the diazaselenurane **12** were also used as the catalysts for the comparison. The initial rate values in Table 1 clearly indicate that all the compounds show much less activity compared to that of the ebselen. However, most of them act as better GPx mimics than the spirodiazaselenurane **12** having aromatic substituents directly attached to the nitrogen atoms. Catalytic activities of these compounds are highly dependent on the nature of the substituent attached to the nitrogen atoms. Selenides **16**–**17** with pyridylmethyl substituents showed good activities in the reduction of peroxides. Particularly, compound **17** showed the highest activity (two times than that of compound **12**) among all the selenides **15**–**21**. Other heterocyclic-based selenides such as **18** and **19** exhibited moderate catalytic activities.

**Table 1:** Initial rates for the reduction of H_2_O_2_ in the presence of catalysts **ebselen**, **12**, **15**–**21** and **29**–**35**.

Compd	v_0_ (μM·min^−1^) ^[a]^	Compd	v_0_ (μM·min^−1^) ^[a]^
**Ebselen**	98.0 ± 3.13	**12**	35.6 ± 1.25
**15**	37.3 ± 1.77	**29**9	51.8 ± 2.91
**16**	65.7 ± 2.06	**30**	77.5 ± 3.99
**17**	74.1 ± 2.48	**31**	79.4 ± 2.87
**18**	52.8 ± 1.81	**32**	58.7 ± 0.95
**19**	42.3 ± 2.14	**33**	50.2 ± 1.80
**20**	54.8 ± 0.76	**34**	56.2 ± 1.53
**21**	50.7 ± 0.79	**35**	32.8 ± 0.65

^[a]^ The reactions were carried out in phosphate buffer (100 mM, pH 7.5) at 20 °C. Catalyst: 80.0 µM; glutathione reduced 2.0 mM; NADPH: 0.4 mM; glutathione disulfide reductase 1.7 unit·mL^−1^ peroxide: 1.6 mM. The initial rates were corrected for the background reaction between peroxide and thiol.

However, replacement of benzylic substituents with aliphatic side chains (compounds **20**–**21**) reduces the activity considerably, as shown in Table 1. The initial rates for compound **20** and **21** (54.8 and 50.7 μM·min^−1^, respectively) are much less than that of selenides **16**–**17** (65.7 and 74.1 μM·min^−1^, respectively). A similar trend in the activity was noticed for the spirodiazaselenuranes **29**–**35** as well. Compounds **30** and **31** with pyridylmethyl moieties were the most active compounds, with initial rates of 77.5 and 79.4 μM·min^−1^, respectively. Other spiro compounds reduce the H_2_O_2_ with moderate activity in the presence of thiol. Interestingly, Table 1 shows that except compound **35**, all the diaryl selenides **15**–**21** and spirodiazaselenuranes **29**–**35** have higher initial rates for the reduction of peroxides than that of the spirodiazaselenurane **12**. This clearly indicates that replacement of aromatic ring by benzylic, pyridylmethyl and heterocyclic substituents increases the antioxidant activity significantly. The GPx-like activity of these compounds was also found to be dependent on the nature of the peroxides. Although these compounds show good catalytic activity in the presence of H_2_O_2_ as substrate, no such significant activity was observed when other peroxides such as *t*-BuOOH or Cum-OOH were used as the substrate in the reactions.

**Scheme 4:**
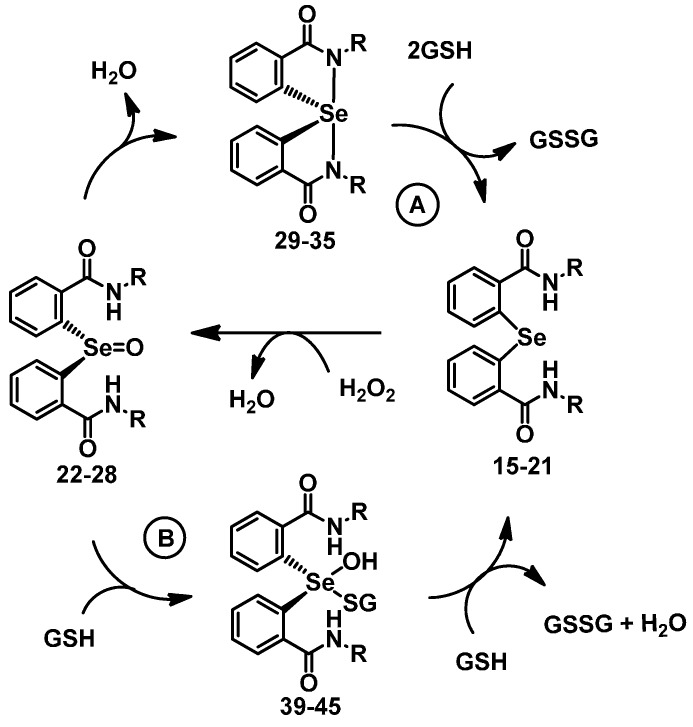
Proposed mechanism for GPx-like activity of compounds **15**–**35**.

The catalytic mechanism of spirodiazaselenuranes **29**–**35** for the reduction of peroxide was studied using ^77^Se-NMR spectroscopy. It was found that substitution of the aromatic ring with benzylic (**29**), pyridylmethyl (**30**–**31**), heterocyclic (**32**–**33**) and aliphatic chains (**34**–**35**) do not alter the catalytic mechanism [21]. Diaryl selenide is the active species, which reduces H_2_O_2_ to water and is oxidized to the corresponding selenoxide intermediate. Reaction of spirodiazaselenuranes **29**–**35** with GSH leads to the reductive cleavage of the Se-N bonds producing the corresponding selenides **15**–**21**, which then react with H_2_O_2_ to generate the corresponding selenoxides **22**–**28**. Subsequent elimination of a water molecule regenerates the spirodiazaselenuranes **29**–**35** (path A, Scheme 4) completing the catalytic cycle. Cycle A is the major pathway at higher peroxide concentration. However, as it is shown earlier [22], additional pathway (path B, Scheme 4) involving the intermediates **39**–**45** is observed at high GSH concentration. Compounds **39**–**45** may be produced by the nucleophilic attack of the GSH at the selenium centre of selenoxides **22**–**28**. Subsequent reaction with second equivalent of GSH regenerates the selenides **15**–**21**. The catalytic cycle clearly indicates that the selenide is mainly responsible for the reduction of H_2_O_2_ to water. As the benzylic and aliphatic substituents reduce the stability of Se-N bonds in the spiro compounds, the spirodiazaselenuranes **29**–**35** readily convert to the corresponding selenides **15**–**21** in the presence of GSH. Therefore, the rapid formation of the selenide species enhances the catalytic activity significantly compared to that of the spirodiazaselenurane **12**, in which the Se-N bonds are relatively more stable due to the presence of phenyl substituents. Therefore, facile cleavage of Se-N bonds in the spirodiazaselenuranes plays an important role in the GPx-like activity.

### 2.4. Inhibition of Peroxynitrite-Mediated Nitration and Oxidation

Peroxynitrite (PN, ONOO^−^) is a powerful nitrating and oxidizing agent, which causes serious damage to several proteins and biomolecules [26,27]. Peroxinitrite is produced *in vivo* by diffusion controlled reaction of nitric oxide (NO) and the superoxide radical (O_2_•‾) [28,29]. During inflammation, the production of PN increases, which subsequently, induces DNA damage [30,31], lipid peroxidation in biomembranes and lipoproteins [32,33]. Peroxynitrite can also inactivate a variety of enzymes by nitrating the tyrosine residue generating 3-nitrotyrosine (3-NT) [34,35,36]. Therefore, inhibition of the PN-mediated nitration and oxidation reaction is very essential. Sies *et al*., have reported several sulphur and selenium containing small molecules which are efficient inhibitor of PN-mediated nitration and oxidation reactions [37]. As many of the selenides and spirodiazaselenuranes react effectively with peroxides, we extended our work to investigate the effect of different substituents on the PN-scavenging antioxidant activity. In the presence of PN, diaryl selenides readily produce the corresponding selenoxides inhibiting the PN-mediated nitration and oxidation reactions [38,39].

**Scheme 5:**
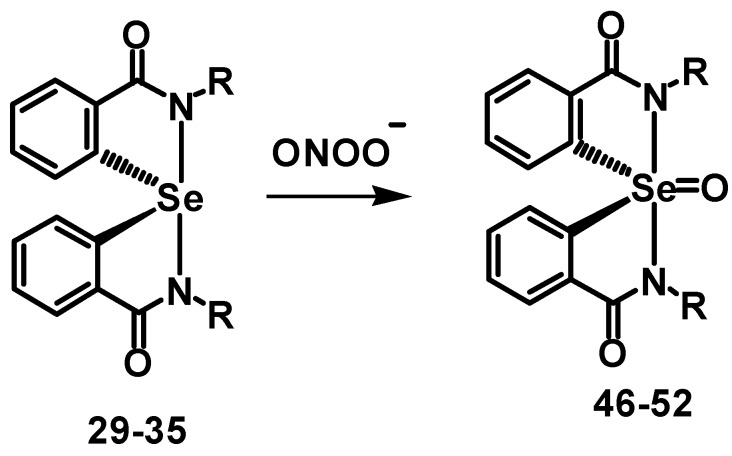
Proposed mechanism for GPx-like activity of compounds **23**–**46**.

**Figure 3:**
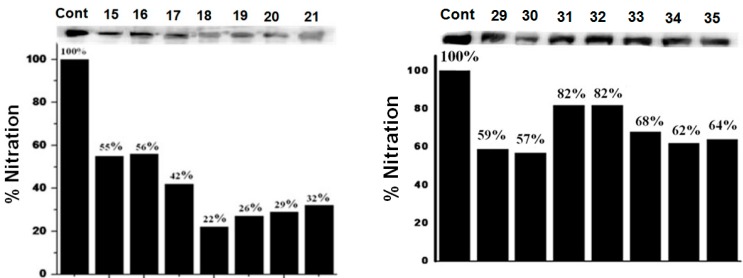
Immunoblots for the inhibition of PN mediated nitration of BSA by diaryl selenides **15**–**21** and spirodiazaselenuranes **29**–**35**. BSA (100 mM) was incubated with inhibitor (166 µm) and PN (1.2 mM) for 30 min at 20 °C and then subjected to gel electrophoresis.

To understand the effect of diaryl selenides **15**–**21** and spirodiazaselenuranes **29**–**35** against PN, the PN-scavenging activity in PN-mediated nitration of tyrosyl residues in bovine serum albumin (BSA) was studied [40,41]. The inhibition of nitration of tyrosine residues was followed by immunoblotting methods using antibody against 3-nitro-l-tryrosine. BSA (100 mM) was treated with PN (1.2 mM) and selenium compounds (166 µM) as inhibitor, and the reaction mixture was kept for incubation at 20 °C for 20 min. The relative inhibitory activities of selenides **15**–**21** are summarized in Figure 3. It is observed from the percentage of nitration that all the selenides are very potent inhibitor of PN- mediated nitration reaction. Particularly, compound **18** with pyridine substituent at the nitrogen atom shows almost 80% inhibition. Other diaryl selenides such as **19** with thiadiazole ring and compounds **20**–**21** with aliphatic side chains also were found to be very effective inhibitors for the PN-mediated nitration reaction. Unlike GPx-like activity, compounds **16** and **17** with pyridylmethyl exhibit relatively less inhibition compared to that of the other selenides. The inhibition of PN-mediated nitration of BSA by spirodiazaselenuranes **29**–**35** were also studied and shown in Figure 3. As our expectation, compounds **29**–**35** show very less inhibition than that of the corresponding selenides **15**–**21**. Compound **30** was found to be the most active inhibitor among all the spirodiazaselenuranes **29**–**35** with 43% inhibition. A significant inhibition of PN-mediated nitration (~20%–50%) by the spiro compounds **29**–**35** clearly indicates that the selenium centre in the spirodiazaselenurane undergoes further oxidation in the presence of PN producing the selenurane oxides **46**–**52** (Scheme 5).

**Scheme 6:**
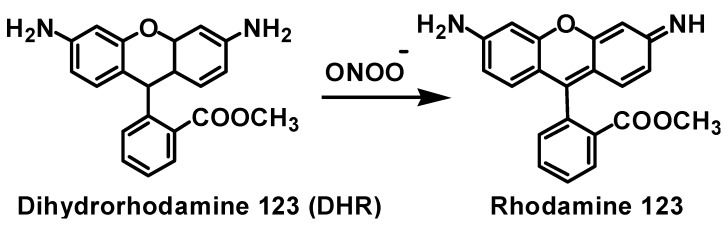
Peroxynitrite-mediated oxidation of dihydrorhodamine 123 (DHR) to rhodamine 123.

PN is also known as a strong oxidizing agent and it oxidizes various biomolecules. Ebselen is an efficient scavenger of PN [42]. Therefore, we have studied the PN-mediated oxidation of dihydrorhodamine 123 (DHR) to rhodamine 123 (Scheme 6) in the presence of the selenides **15**–**21** and spirodiazaselenuranes **29**–**35**. Rhodamine 123 is a highly fluorescent compound and the increase in the fluorescent intensity by PN-mediated oxidation was monitored using fluorescence spectrometry. The concentration of the selenium compounds required for the 50% inhibition of the oxidation reaction are represented as IC_50_ values and are listed in Table 2 for the selenides and the spirodiazaselenuranes.

The IC_50_ value of ebselen for inhibiting the PN-mediated oxidation of DHR to rhodamine is very low (0.92 µM), which suggests that ebselen is an efficient scavenger of PN. However, diaryl selenides and spirodiazaselenuranes exhibit much less scavenging activity than that of ebselen. Although these compounds are poorer PN scavengers than ebselen, the inhibition is much higher compared to that of the spirodiazaselenurane **12**. Particularly, the selenides **16**–**18** having heterocyclic pyridine substituents inhibit the PN-mediated oxidation significantly, with IC_50_ values of 5.55, 4.23 and 2.50 µM, respectively (Table 2). Similar to the PN-mediated BSA nitration assay, selenide **18** showed the highest scavenging activity in DHR oxidation reaction. Other selenides were also found to inhibit the PN-mediated oxidation moderately. The significant PN scavenging activity of all the spirodiazaselenuranes **29**–**35** than that of compound **12** also proves that selenium centre undergoes further oxidation in the presence of excess PN producing the corresponding selenurane oxides. Interestingly, the heterocyclic pyridine substituted selenides (**16**–**18**) and spirodiazaselenuranes (**30**–**32**) were found to be excellent GPx mimics and also effective inhibitors for the PN-mediated nitration and oxidation reactions. This strongly indicates that the stability of the Se-N bonds in the spiro compounds and the antioxidant activity of diaryl selenides and spirodiazaselenuranes are greatly influenced by the heterocylic substituents present at the nitrogen atoms.

**Table 2:** Inhibition of PN-mediated oxidation reaction of dihydrorhodamine 123 by **ebselen**, compound **12**, selenides **15**–**21** and spirodiazaselenuranes **29**–**35**.

Compd	IC_50_ µM ^[a]^	Compd	IC_50_ µM ^[a]^
**ebselen**	0.92 ± 0.04	**12**	72.56 ± 1.28
**15**	8.83 ± 0.39	**29**	12.83 ± 0.49
**16**	5.55 ± 0.39	**30**	20.84 ± 0.70
**17**	4.23 ± 0.27	**31**	34.81 ± 0.15
**18**	2.50 ± 0.18	**32**	33.59 ± 0.30
**19**	18.33 ± 0.23	**33**	29.79 ± 0.16
**20**	16.37 ± 0.07	**34**	23.83 ± 0.96
**21**	21.30 ± 0.17	**35**	39.59 ± 0.26

^[a]^ Assay conditions: Dihydrorhodamine 123 (DHR; 0.1 mM), peroxynitrate (0.69 mM), and inhibitors (variable) in sodium phosphate buffer (100 mM, pH 7.5) with DTPA (100 mM) at 23 °C. Percentage of inhibition at 200 mM concentrations were in the range 20%–30%.

**Scheme 7:**
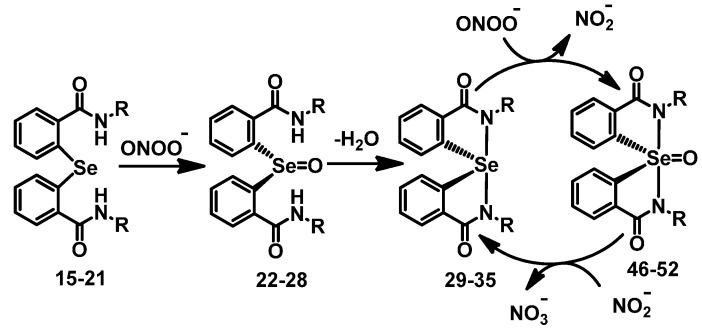
Proposed mechanism for the reduction and isomerization of peroxynitrite by the diaryl selenides and spirodiazaselenuranes.

From the experimental evidences it is observed that, although replacement of the aromatic rings with benzylic/aliphatic substituents enhances the PN scavenging activity, the catalytic cycle for the isomerisation of PN remains unaltered. Similar to the previous reports [22,43], the diaryl selenides (**15**–**21**) first react with one equivalent of PN to produce the corresponding selenoxides (**22**–**28**), which after elimination of one water molecule generate the corresponding spirodiazaselenuranes (**29**–**35**). Subsequent reaction of these spiro compounds with second equivalent of PN leads to the overoxidation of selenium centres producing the corresponding selenurane oxides (**46**–**52**). However, a redox cycle was formed between the spiro compounds and their oxides, when the later compounds react with nitrite, produced in the reaction mixture to regenerate the spirodiazaselenuranes along with the nitrate (NO_3_^−^). No regeneration of the selenides was observed in the ^77^Se-NMR spectra. Therefore, in the presence of PN and nitrite, the spirodiazaselenuranes act as catalysts for the isomerizaion of PN to nitrate (Scheme 7). The selenides act as pro-catalyst in the isomerisation reaction as they are not directly involved in the redox cycle.

## 3. Experimental Section

### 3.1. General Procedures

Selenium powder was purchased from Aldrich Chemical Co. (St. Louis, MO, USA). Acetonitrile and dichlomethane were dried over P_2_O_5_. All other chemicals were of the highest purity available. All experiments were carried out under anhydrous and anaerobic conditions using standard Schlenk techniques for the synthesis. Due to unpleasant odors of several of the reaction mixtures involved, most manipulations were carried out in a well-ventilated fume hood. ^1^H- (400 MHz), ^13^C- (100.56 MHz), and ^77^Se- (76.29 MHz) NMR spectra were obtained at room temperature on a 400 MHz NMR spectrometer (Bruker Optik, Ettlingen, Germany). Chemical shifts are quoted with respect to SiMe4 as internal (^1^H and ^13^C), and Me_2_Se as external (^77^Se) standards. A Lambda 5 UV/Vis spectrophotometer (Perkin–Elmer, Waltham, MA, USA) was used to measure GPx activity. The melting points (uncorrected) of the compounds were determined in an open capillary with a B-540 Melting Point apparatus (BOchi Labortechnik AG, Flawil, Switzerland). Mass spectral studies were carried out on a Q-TOF micro mass spectrometer (Waters Inc., Milford, MA, USA) with ESI MS mode analysis. Thin-layer Chromatography analyses were carried out on precoated silica gel plates (Merck, Darmstadt, Germany), and spots were visualized by UV irradiation. Column chromatography was performed on glass columns loaded with silica gel or on an automated flash chromatography system (Biotage, Uppsala, Sweden) by using preloaded silica cartridges.

### 3.2. GSH–GSSG Coupled Assay

The GPx activity was followed spectrophotometrically. The initial reduction rates were calculated from the rate of NADPH oxidation at 340 nm in a GSH assay. Each initial rate was measured at least three times and calculated from the first 5%–10% of the reaction by using 6.22 mM^−1^·cm^−1^ as the molar extinction coefficient for NADPH. For the peroxidase activity, the rates were corrected for the background reaction between peroxide and thiol.

### 3.3. Peroxynitrite-Mediated Oxidation Assay

Peroxynitrite-mediated oxidation of dihydrorhodamine 123 (DHR) was followed as suggested in the literature with minor modifications [44]. Fluorescence intensity was measured using a Perkin-Elmer LS 50B luminescence spectrometer with excitation and emission wavelengths of 500 and 526 nm, respectively. The stock solution of DHR in dimethylformamide (DMF) was purged with nitrogen and stored at −20 °C. The working solutions of DHR and peroxynitrite were kept on an ice bath. The assay mixture contained DHR (0.50 μM) and peroxynitrite (1.0 μM) in 100 mM sodium phosphate buffer of pH 7.4 with 100 μM DTPA and variable inhibitor concentrations. The fluorescence intensity from the reaction of DHR with PN was set as 100%, and the intensity after the addition of various inhibitor amounts was expressed as the percentage of that observed in the absence of inhibitors. The final fluorescence intensities were corrected for background reactions. The inhibition plots were obtained using Origin 6.1 software, and these plots were used for calculating the IC_50_ values of different inhibitors.

### 3.4. Inhibition of PN-Mediated Nitration of BSA

For bovine serum albumin (BSA), the nitration was performed by the addition of PN (1.2 mm) to BSA (100 mm) in 0.5 m phosphate buffer of pH 7.0 with DTPA (0.1 mm) at 20 °C. After the addition of PN, the final pH was maintained below 7.5. The reaction mixture was incubated for 20 min at 20 °C. Similarly, the reactions of BSA with PN were performed in the presence of selenides and spiro compounds (166 µm) as inhibitors. Upon performing the reactions, the mixture was denatured by boiling at 100 °C for 5 min in the presence of sample loading dye and subjected to polyacrylamide gel electrophoresis and Western blot analyses.

### 3.5. Electrophoretic Analysis

Gel was prepared with 10% and 15% polyacrylamide with 6% stacking gel for BSA. The gel was run in the running buffer of pH 8.8 with glycine and sodium dodecyl sulfate (SDS). After separating the proteins, the gel was analyzed by Western blot experiments. The proteins were transferred to a polyvinylidene fluoride (PVDF) membrane and the nonspecific binding sites were blocked by 5% nonfat skimmed milk in PBST (blocking solution) for 2 h. Then the membrane was probed with rabbit polyclonal primary antibody against nitrotyrosine (1:20,000 dilutions) in blocking solution for 2 h followed by incubation with horseradish peroxidase conjugated donkey polyclonal anti-rabbit IgG (1:20,000 dilutions) for another 2 h. The probed membrane was then washed three times with blocking solution with 0.1% Tween 20, and the immunoreactive protein was detected by luminol-enhanced chemiluminiscence (ECL, Amersham, UK).

### 3.6. X-ray Crystallography

X-ray crystallographic studies were carried out on a Bruker CCD diffractometer with graphite-monochromatized MoKa radiation (l = 0.71073 λ) controlled by a Pentium-based PC running on the SMART software package [45]. Single crystals were mounted at room temperature on the ends of glass fibers, and data were collected at room temperature. The structures were solved by direct methods and refined by using the SHELXTL software package [46,47]. All non-hydrogen atoms were refined anisotropically, and hydrogen atoms were assigned idealized locations. Empirical absorption corrections were applied to all structures by using SADABS software [48]. The structures were solved by direct methods (SIR-92) and refined by a full-matrix least-squares procedure on F2 for all reflections (SHELXL-97) [49].

*Crystal data for* **16**: C_26_H_22_N_4_O_2_Se; *Mr* = 503.2; Monoclinic; space group P 2 (1)/*c a* = 9.4004(15), *b* = 28.087(4), *c* = 9.9004(15) Å; α = 90.00(2)°, β = 113.66(2)°, γ = 90.00(2)°, V = 2273.35 Å^3^; *Z* = 4; ρ_calcd_ = 1.36 g·cm^−3^; Mo_K\α_ radiation (λ = 0.71073 Å); T = 296(2) K; *R*_1_, = 0.0709, *wR*_2_ = 0.1532 (I > 2σ(I); *R*_1_ = 0.0429, *wR*_2_ = 0.1011. CCDC number: 1404888.

*Crystal data for* **32**: C_24_H_16_N_4_O_2_Se; *Mr* = 471.37; Monoclinic; space group *C2/c a* = 18.278(11), *b* = 14.734(9), *c* = 9.1876(4) Å; α = 90.00°, β = 118.414(2)°, γ = 90.00°; V = 2176.32 Å^3^; *Z* = 4; ρ_calcd_ = 1.44g cm^−3^; Mo_K\α_ radiation (λ = 0.71073 Å); T = 296(2) K; *R*_1_, = 0. 0.0257, *wR*_2_ = 0.1565 (I > 2σ(I); *R*_1_ = 0.0392, *wR*_2_ = 0.1194. CCDC number: 1404889.

### 3.7. General Synthesis of Selenides

A solution of corresponding amine (2.8 mmol) in dry acetonitrile (10 mL) was added dropwise to a mixture containing acid chloride (1.40 mmol) and the reaction mixture was stirred for additional 6 h at room temperature. The solvent was removed under vacuum to obtain a light yellow solid, which was then purified by flash chromatography using petroleum ether/ethyl acetate as the eluents.

*Synthesis of 2,2′-selenobis(N-(4-methoxybenzyl)benzamide)* (**15**): Brown solid with 89% yield. m.p: 182–183 °C. ^1^H-NMR (DMSO-*d*_6_, ppm): δ 6.79–6.80 (d, *J* = 6.78 Hz, 2H), 6.82–6.91 (d, *J* = 6.8 Hz, 2H), 6.92–6.94 (d, *J* = 6.92 Hz, 2H), 7.19–7.23 (m, 4H), 7.23–7.26 (d, *J =* 7.24 Hz, 2H), 7.28–7.29 (m, 2H), 7.30–7.39 (m, 2H), 8.54–8.54 (d, *J* = 8.4 Hz, 2H), 4.32 (s, 4H), 3.89 (s, 6H). ^13^C-NMR (DMSO-*d*_6_, ppm): δ 42.5, 56.0, 114.4, 114.7, 127.8, 128.5, 131.0, 131.5, 132.0, 133.3, 133.8, 135.8, 137.2, 160.1, 165.6. ^77^Se-NMR (DMSO): 426.7. ESI-MS, (*m*/*z*) Calcd, C_30_H_28_N_2_O_4_Se: 558.4, found: 559.1 [M + H]^+^.

*Synthesis of 2,2′-selenobis(N-(pyridin-2-ylmethyl)benzamide)* (**16**); White solid with 88% yield. ^1^H-NMR (CDCl_3_, ppm): δ 6.82–6.84 (d, *J* = 6.82 Hz, 2H), 7.15–7.16 (d, *J* = 7.14 Hz, 2H), 7.31–7.38 (m, 2H), 7.48–7.49 (d, *J* = 7.4 Hz, 2H), 7.55–7.57 (d, *J* = 7.55 Hz, 4H), 7.80–7.81 (d, 2H), 7.88–7.91 (m, 2H), 8.16–8.18, (m, 2H), 8.37–8.39 (d, *J* = 8.4 Hz, 2H), 4.49–4.50 (d, *J* = 7.0 Hz, 2H). ^13^C-NMR (DMSO-*d*_6_, ppm): δ 43.5, 121, 123, 123, 124, 127, 128, 131, 133, 134, 137, 138, 138, 149, 149, 154, 159, 169. ^77^Se-NMR (DMSO): 422. ESI-MS (*m*/*z*) Calcd, C_26_H_22_N_4_O2Se: 502.4, found: 525.1 [M + Na]^+^.

*Synthesis of 2,2′-selenobis(N-(pyridin-3-ylmethyl)benzamide)* (**17**): Yellowish solid with 87% yield. ^1^H-NMR (CDCl_3_, ppm): δ 6.80–6.82 (d, *J =* 6.81 Hz, 2H), 7.07–7.08 (d, *J* = 7.0 Hz, 2H), 7.15–7.16 (d, *J =* 7.15 Hz, 2H), 7.31–7.38 (m, 2H), 7.48–7.49 (d, *J* = 7.4 Hz, 2H), 7.55–7.57 (d, *J =* 7.55 Hz, 4H), 7.80–7.81 (d, 2H), 7.88–7.91 (m, 2H), 8.16–8.18, (m, 2H), 4.49–4.50 (d, *J =* 6.8 Hz, 2H). ^13^C-NMR (DMSO-*d*_6_, ppm): δ 43.5, 121, 123, 123, 124, 127, 128, 131, 133, 134, 137, 138, 138, 149, 149, 154, 159, 169. ^77^Se-NMR (DMSO): 416. ESI-MS (*m*/*z*) Calcd, C_26_H_22_N_4_O_2_Se: 503.2, found: 525.7 [M + Na]^+^.

*Synthesis of 2,2′-selenobis(N-(pyridin-3-yl)benzamide)* (**18**): White solid with 93% yield. ^1^H-NMR (DMSO-*d*_6_, ppm): δ 7.32–7.33 (d, *J* = 7.31 Hz, 2H), 7.34–7.45 (m, 2H), 7.82–7.85 (m, 2H), 7.92–7.93 (d, *J* = 7.2 Hz, 2H), 7.94–7.96 (d, *J* = 7.4 Hz, 2H), 8.58–8.61 (d, *J =* 7.6 Hz, 2H), 8.93 (d, *J* = 8.4 Hz, 2H), 8.16–8.18, (m, 2H), 11.4, (s, 2H). ^13^C-NMR (CDCl_3_, ppm): δ 124.8, 126.6, 130.7, 131.1, 133.9, 135.0, 135.1, 135.2, 138.0, 144.4, 146.6, 165.6. ^77^Se-NMR (DMSO): 429. ESI-MS (*m*/*z*) Calcd, C_24_H_18_N_4_O_2_Se: 474.4, found: 500.7 [M + Na]^+^.

*Synthesis of 2,2′-selenobis(N-(5-phenyl-1,3,4-thiadiazol-2-yl)benzamide)* (**19**): Yellowish solid with 78% yield. ^1^H-NMR (DMSO-*d*_6_, ppm): δ 7.28–7.36 (m, 4H), 7.49–7.51 (d, *J* = 8.4 Hz, 2H), 7.58–7.59 (d, *J =* 7.6 Hz, 4H), 7.67–7.68 (d, *J =* 8.0 Hz, 4H), 7.73–7.75 (d, *J =* 7.73 Hz, 2H), 7.80–7.82 (d, *J =* 7.6 Hz, 2H), 7.91–7.93 (d, *J* = 7.0 Hz, 2H). ^13^C-NMR (DMSO-*d*_6_, ppm): δ 124.8, 126.6, 130.7, 131.1, 133.9, 135.0, 135.1, 135.2, 138.0, 144.4, 146.6, 165.6. ^77^Se-NMR (DMSO): 465. ESI-MS (*m*/*z*) Calcd, C_30_H_20_N_6_O_2_S_2_Se: 640.4, found: 640.7 [M^+^].

*Synthesis of 2,2′-selenobis(N-(1-hydroxy-2-methylpropan-2-yl)benzamide)* (**20**): White solid with 88% yield. ^1^H-NMR (CDCl_3_, ppm): δ 1.24 (s, 12H), 3.62 (s, 4H), 7.31–7.37 (m, 4H), 7.51–7.53 (d, *J =* 7.51 Hz, 2H), 7.58–7.61 (m, 2H), 7.95–7.99 (m, 4H). ^13^C-NMR (CDCl_3_ ppm): δ 23.0, 56.0, 68.3, 120.4, 125.5, 125.5, 128.1, 129.2, 130.7, 131.1, 131.7, 132.3, 134.6, 139.9, 170.5. ^77^Se-NMR (CHCl_3_): 410, ESI-MS, (*m*/*z*) Calcd, C_22_H_28_N_2_O_4_Se: 463.4, found: 487.2 [M + Na]^+^.

*Synthesis of 2,2′-selenobis(N-(2-(diethylamino)ethyl)benzamide)* (**21**): White solid with 73% yield. ^1^H-NMR (CDCl_3_, ppm): δ 1.18 (m, 6H), 2.29–2.98 (m, 4H), 3.00–3.07 (m, 4H), 3.65–3.66 (d, *J =* 7.0 Hz, 4H), 7.04 (s, 2H), 7.24–7.26 (d, *J =* 7.0 Hz, 4H), 7.28–7.28 (d, *J =* 7.2 Hz, 4H), 7.32–7.33 (d, *J =* 7.0 Hz, 4H), 7.33–7.35, (d, *J =* 7.4 Hz, 4H), 8.41, (s, 2H). ^13^C-NMR (CDCl_3_ ppm): δ 10.01, 36.2, 47.3, 51.65, 127.8, 128.6, 129.7, 131.0, 131.5, 132.6, 133.3, 134.9, 137.7, 169.5. ^77^Se-NMR (MeOH): 429. ESI-MS (*m*/*z*) Calcd. C_26_H_38_N_2_O_4_Se: 518.0, found: 520.7 [M + H]^+^.

### 3.8. General Synthesis of Spirodiazaselenuranes

To a stirred solution of the selenide (0.44 mmol) in dry CH_2_Cl_2_ (5 mL), hydrogen peroxide (0.44 mmol) was added. The reaction mixture was stirred for 5 h at room temperature and the solvent was evaporated under vacuum. The compounds were purified by column chromatography using silica gel with petroleum ether and ethyl acetate as the eluents.

*Synthesis of 2,2′-bis(4-methoxybenzyl)-1λ^4^-1,1′-spirobi[benzo[d][1,2]selenazole]-3,3′(2H,2′H)-dione* (**29**): white crystalline solid with 95% yield. m.p: 203–204 °C. ^1^H-NMR (DMSO-*d*_6_, ppm): δ 6.80–6.83 (d, *J =* 6.81 Hz, 2H), 6.93–6.95 (d, *J =* 6.93 Hz, 2H), 7.31–7.33 (d, *J =* 7.31 Hz, 2H), 7.41–7.43 (d, *J =* 7.6 Hz, 2H), 7.51–7.53 (d, *J =* 7.51 Hz, 2H), 7.55–7.57 (m, 4H), 7.67–7.92 (m, 2H), 3.74 (s, 4H), 3.90, (s, 6H). ^13^C-NMR (DMSO-*d*_6_, ppm): δ 41.6, 55.24, 113.9, 126.0, 127.7, 130.1, 130.6, 131.1, 132.4, 132.9, 135.0, 136.3, 158.2. 165.6. ^77^Se-NMR (DMSO): 554. ESI-MS (*m*/*z*) Calcd, C_30_H_26_N_2_O_4_Se: 558.6, found: 559.1 [M + H]^+^.

*Synthesis of 2,2′-bis(pyridin-2-ylmethyl)-1λ^4^-1,1′-spirobi[benzo[d][1,2]selenazole]-3,3′(2H,2′H)-dione* (**30**): Brown solid with 83% yield. ^1^H-NMR (CDCl_3_, ppm): δ 7.13–7.14 (d, *J =* 5.2 Hz, 2H), 7.15–7.16 (d, *J =* 4.8 Hz, 2H), 7.27–7.28 (d, *J =* 7.2 Hz, 2H), 7.30–7.31 (d, *J =* 7.2 Hz, 2H), 7.33–7.34 (d, *J =* 7.2 Hz, 2H), 7.39–7.41 (m, 2H), 7.60–7.70 (m, 2H), 8.44–8.45, (d, *J* = 4.6 Hz, 2H), 4.62–4.63 (d, *J =* 4.8 Hz, 4H). ^13^C-NMR (DMSO-*d*_6_, ppm): δ 45.3, 122.6, 122.7, 128.0, 129.0, 131.6, 131.9, 135.1, 137.2, 137.8, 149.3, 156.6, 168.7. ^77^Se-NMR (DMSO): 561. ESI-MS (*m*/*z*) Calcd, C_26_H_20_N_2_O_4_Se: 503.4, found: 523.1 [M + Na]^+^.

*Synthesis of 2,2′-bis(pyridin-3-ylmethyl)-1λ^4^-1,1′-spirobi[benzo[d][1,2]selenazole]-3,3′(2H,2′H)-dione* (**31**): Yellowish solid with 80% yield. ^1^H-NMR (CDCl_3_, ppm): δ 7.09–7.10 (d, *J =* 6.8 Hz, 2H), 7.26–7.27 (d, *J =* 7.0 Hz, 2H), 7.35–7.37 (d, *J =* 7.6 Hz, 2H), 7.38–7.39 (d, *J =* 7.4 Hz, 2H), 7.75–7.76 (d, *J =* 7.6 Hz, 2H), 7.77–7.78 (m, 2H), 8.39–8.40 (d, *J =* 7.0 Hz, 2H), 8.05–8.41 (m, 2H), 8.53–8.54, (d, *J =* 7.6 Hz, 2H), 4.62–4.63 (d, *J =* 7.0 Hz, 2H). ^13^C-NMR (CDCl_3_, ppm): δ 43.56, 121.9, 123.1, 123.5, 124.2, 128.6, 131.6, 133.1, 134.8, 137.8, 138.1, 149.3, 169.1. ^77^Se-NMR (CD_3_OD+DMSO): 553. ESI-MS (*m*/*z*) Calcd, C_26_H_20_N_2_O_4_Se: 500.5, found: 523.5 [M + Na]^+^.

*Synthesis of 2,2′-di(pyridin-3-yl)-1λ^4^-1,1′-spirobi[benzo[d][1,2]selenazole]-3,3′(2H,2′H)-dione* (**32**): Brownish solid with 97% yield. ^1^H-NMR (DMSO-*d*_6_, ppm): δ 7.36–7.39 (m, 2H), 7.64–7.67 (m, 2H), 7.74–7.77 (d, *J =* 7.0 Hz, 2H), 7.78–7.83 (d, *J =* 7.6 Hz, Hz, 2H), 8.22–8.25 (m, 2H), 8.28–8.30 (d, *J =* 7.4 Hz, 2H), 8.41–8.41 (d, *J =* 7.0 Hz, 2H), 8.42–8.43 (d, *J =* 7.6 Hz, 2H). ^13^C-NMR (CDCl_3_, ppm): δ 124.8, 126.6, 130.7, 131.1, 133.9, 135.0, 135.1, 135.2, 138.0, 144.4, 146.6, 165.6. ^77^Se-NMR (DMSO): 581. ESI-MS (*m*/*z*) Calcd, C_24_H_16_N_4_O_2_Se: 472.4, found: 474.0 [M + H]^+^.

*Synthesis of 2,2′-bis(5-phenyl-1,3,4-thiadiazol-2-yl)-1λ^4^-1,1′-spirobi[benzo[d][1,2]selenazole]-3,3′(2H,2′H)-dione* (**33**): Yellowish solid with 93% yield. ^1^H-NMR (DMSO-*d*_6_, ppm): δ 7.39–7.42 (d, *J =* 7.0 Hz, 2H), 7.42–7.47 (m, 2H), 7.48–7.51 (d, *J =* 7.6 Hz, 2H), 7.55–7.56 (m, 2H), 7.65–7.67 (d, *J =* 7.6 Hz, 2H), 7.71–7.73 (d, *J =* 7.0 Hz, 2H), 7.77–7.78 (d, *J =* 7.6 Hz, 2H), 7.80–7.81 (d, *J =* 7.6 Hz, 2H), 7.81–7.81 (d, *J =* 7.6 Hz, 2H). ^13^C-NMR (CDCl_3_, ppm): δ 127.1, 129.5, 130.0, 130.1, 130.3, 130.4, 134.0, 136.2, 142.5, 144.4, 146.6, 169.9. ^77^Se-NMR (DMSO): 717. ESI-MS (*m*/*z*) Calcd, C_30_H_18_N_6_O_2_S_2_Se: 638.4, found: 640.6 [M + H]^+^.

*Synthesis of 2,2′-bis(1-hydroxy-2-methylpropan-2-yl)-1λ^4^-1,1′-spirobi[benzo[d][1,2]selenazole]-3,3′(2H,2′H)-dione* (**34**): Yellowish solid with 78% yield. ^1^H-NMR (CDCl_3,_ ppm): δ 1.33 (s, 12H), 3.63 (s, 4H), 7.26–7.36 (m, 2H), 7.41–7.75 (m, 2H), 7.52–7.55 (m, 2H), 7.88–7.94 (m, 4H). ^13^C-NMR (CDCl_3_ ppm): δ 24.65, 56.9, 69.1, 120.2, 126.0, 126.8, 128.4, 128.9, 131.1, 131.1, 131.7, 132.3, 134.9, 138.4, 169.1. ^77^Se-NMR (CDCl_3_): 685, ESI-MS, (*m*/*z*) Calcd, C_22_H_26_N_2_O_4_Se: 462.4, found: 487.0 [M + Na]^+^.

*Synthesis of 2,2′-bis(2-(diethylamino)ethyl)-1λ^4^-1,1′-spirobi[benzo[d][1,2]selenazole]-3,3′(2H,2′H)-dione* (**35**): Reddish solid with 93% yield. ^1^H-NMR (CDCl_3_, ppm): δ 1.16–1.20 (t, *J =* 7.6 Hz, 6H), 2.85–2.90 (m, *J =* 7.2 Hz, 4H), 3.01–3.04 (t, *J =* 6.8 Hz, 4H), 4.11–4.15 (d, *J =* 6.8 Hz, 4H), 7.04 (s, 2H), 7.37–7.41 (m, 4H), 7.53–7.55 (d, *J =* 7.6 Hz, 4H), 7.58–7.62 (m, 4H), 8.00–8.02, (d, *J =* 7.6 Hz, 4H), ^13^C-NMR (CDCl_3_ ppm): δ 10.80, 41.3, 47.2, 51.2, 117.0, 120.8, 124.5, 125.9, 126.9, 130.6, 132.3, 141.7, 169.5. ^77^Se-NMR (MeOD): 678, ESI-MS (*m*/*z*) Calcd, C_26_H_36_N_4_O_2_Se: 516.0, found: 517.1 [M + H]^+^.

*Synthesis of 2,2′-selenobis(N-ethyl-N-(pyridin-2-ylmethyl)benzamide)* (**36**): NaH (0.80 mmol) was added to a dry THF (5 mL) solution of selenide 16 (0.79 mmol). After 10 min of stirring, iodoethane (0.39 mmol) was added drop wise to the reaction mixture. It was stirred for additional 12 h at room temperature. The precipitate was filtered off and dried to obtain the cream white solid. The compound was purified in column chromatography using petroleum ether and ethyl acetate as eluents. Yield: 82%, ^1^H-NMR (CDCl_3_, ppm): δ 1.25–1.31 (m, 6H), 4.48–4.50 (d, *J =* 6.8 Hz, 4H), 3.17–3.21 (m, 4H), 7.02–7.06 (d, *J* = 7.04 Hz, 2H), 7.18–7.22 (m, 2H), 7.23–7.28 (d, *J = 7.25* Hz, 2H), 7.39–7.42 (t, d, *J =* 8.0 Hz, 2H), 7.69–7.69 (m, 2H), 7.71–7.73 (d, *J =* 7.72 Hz, 2H), 8.52–8.54 (d, *J =* 8.0 Hz, 2H), 8.56–8.57 (d, *J =* 8.4 Hz, 2H). ^13^C-NMR (CDCl_3_, ppm): δ 30.18, 44.00, 55.8, 114.4, 114.6, 115.3, 128.2, 129.1, 1290.6, 130.5, 131.1, 131.8, 134.9, 135.1, 159.3, 168.5. ^77^Se-NMR (CDCl_3_): 370, ESI-MS (*m*/*z*) Calcd, C_30_H_30_N_4_O_2_Se: 558.1, found: 581.0 [M + Na]^+^.

*Synthesis of 2,2′-seleninylbis(N-ethyl-N-(pyridin-2-ylmethyl)benzamide)* (**37**): To a stirred solution of 36 (0.13 mmol) in dry CH_2_Cl_2_ (5 mL), hydrogen peroxide (0.13 mmol) was added. The reaction mixture was stirred for 4 h at room temperature. The solvent was evaporated under vacuum to obtain a dark brown solid, which was purified by column chromatography using petroleum ether and ethyl acetate as eluents. Yield: 76%, ^1^H-NMR (CDCl_3_, ppm): δ 1.25–1.50 (m, 6H), 4.63–4.64 (d, *J =* 5.6 Hz, 4H), 3.20–3.22 (m, 4H), 7.18–7.20 (d, *J =* 7.18 Hz, 2H), 7.27–7.30 (m, 2H), 7.34–7.36 (d, *J =* 7.6 Hz, 2H), 7.63–7.66 (t, *J =* 6.0 Hz, 2H), 7.69–7.73 (m, 2H), 8.06–8.07 (d, *J =* 7.2 Hz, 2H), 8.43–8.45 (d, *J =* 4.8 Hz, 2H), 8.51–8.52 (d, *J =* 4.8 Hz, 2H). ^13^C-NMR (CDCl_3_, ppm): δ 30.17, 46.5, 55.8, 113.4, 114.1, 120.3, 128.2, 129.6, 130.4, 132.2, 135.4, 144.4, 146.3, 135.1, 160.6, 168.1. ^77^Se-NMR (CDCl_3_): 863, ESI-MS (*m*/*z*) Calcd, C_30_H_30_N_4_O_3_Se: 574.6, found: 575.1.0 [M + H]^+^.

*Synthesis of 2,2′-bis(pyridin-2-ylmethyl)-1,2,2′-trihydro-3H,3′H-1λ^6^-1,1'-spirobi[benzo[d][1,2]selenazole]-3,3′-dione 1-oxide* (**38**): To a stirred solution of 16 (0.13 mmol) in dry CH_2_Cl_2_ (5 mL), excess ONOO^−^ (0.13 mmol) was added. The reaction mixture was stirred for 4 h at room temperature and the solvent was evaporated under vacuum to obtain a white brown solid. The compound was purified by column chromatography using petroleum ether and ethyl acetate as eluent. Yield: 76%, ^1^H-NMR (CDCl_3_, ppm): δ 7.55–7.58 (t, *J =* 6.4 Hz, 2H), 7.72–7.74 (m, 4H), 7.86–7.88 (d, *J =* 8.0 Hz, 2H), 8.08–8.12 (d, *J =* 6.8 Hz, 4H), 8.58–8.59 (d, *J =* 5.2Hz, 2H), 8.70 (m, 2H). 4.85–4.87 (d, *J =* 5.6 Hz, 4H) ^13^C-NMR (DMSO-*d*_6_, ppm): δ 42.9, 127.1, 127.9, 130.5, 130.5, 130.6, 131.5, 132.0, 132.2, 134.1, 134.6, 136.3, 142.3, 157.7, 170.1. ^77^Se-NMR (DMSO-*d*_6_) 698, ESI-MS (*m*/*z*) Calcd, C_26_H_20_N4O_3_Se: 516.8, found: 537.9 [M + Na]^+^.

## 4. Conclusions

In conclusion, a series of diaryl selenides and spirodiazaselenuranes containing benzyl, pyridylmethyl, heterocyclic and aliphatic substituents on the nitrogen atoms were synthesized. Detailed experimental studies showed that replacement of the phenyl-substituted/phenyl rings with benzylic/heterocyclic/aliphatic moieties decreases the stability of the Se-N bonds considerably in the spiro compounds. The selenoxide intermediates involved in the cyclization process could be isolated at room temperature. Interestingly, in the presence of excess of peroxynitrite, the spiro compounds undergo further oxidation to produce the corresponding selenurane oxides, which are the first example of stable selenurane oxides containing two nitrogen atoms. The comparison of the GPx-like activity showed that the antioxidant activity increases remarkably upon replacement of the aromatic ring with benzylic/aliphatic substituents. Immediate formation of the selenides from the corresponding spiro compounds by GSH is responsible for enhancement of the activity. In addition to this, PN scavenging studies by inhibiting the PN-mediated oxidation and nitration shows that all the selenides and some of the spirodiazaselenuranes possess high scavenging activity. Particularly, selenides **16**–**18** containing heterocyclic pyridine substituents and their spiro forms **30**–**32** exhibit significant GPx-like and PN scavenging activities.

## Supplementary Materials

Supplementary materials can be accessed at: http://www.mdpi.com/1420-3049/20/07/12959/s1.
