# Supplementary material for: Substituent Effects on the Stability and Antioxidant Activity of Spirodiazaselenuranes
Source: Molecules. 2015 Jul 17;20(7):12959–78. doi: 10.3390/molecules200712959 (PMC6332218; doi:10.3390/molecules200712959)
Supplement: Supplementary file 1 [file molecules-20-12959-s001.pdf]

Supplementary Material

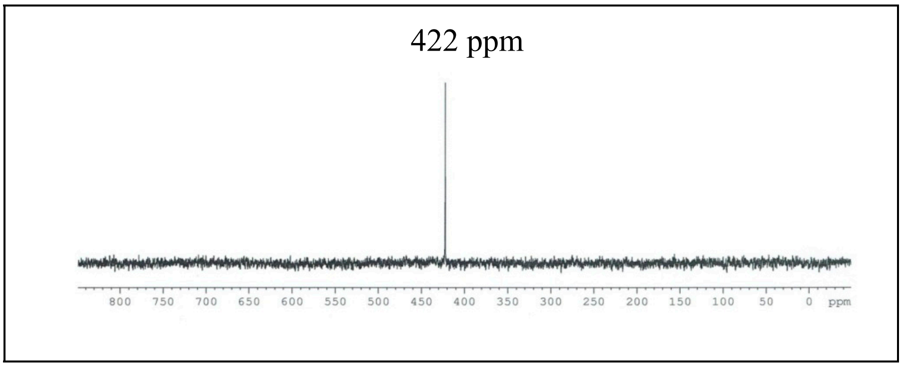

Figure S1. ^{77}Se-NMR spectra of pure diaryl selenide **16** in DMSO-d_6.

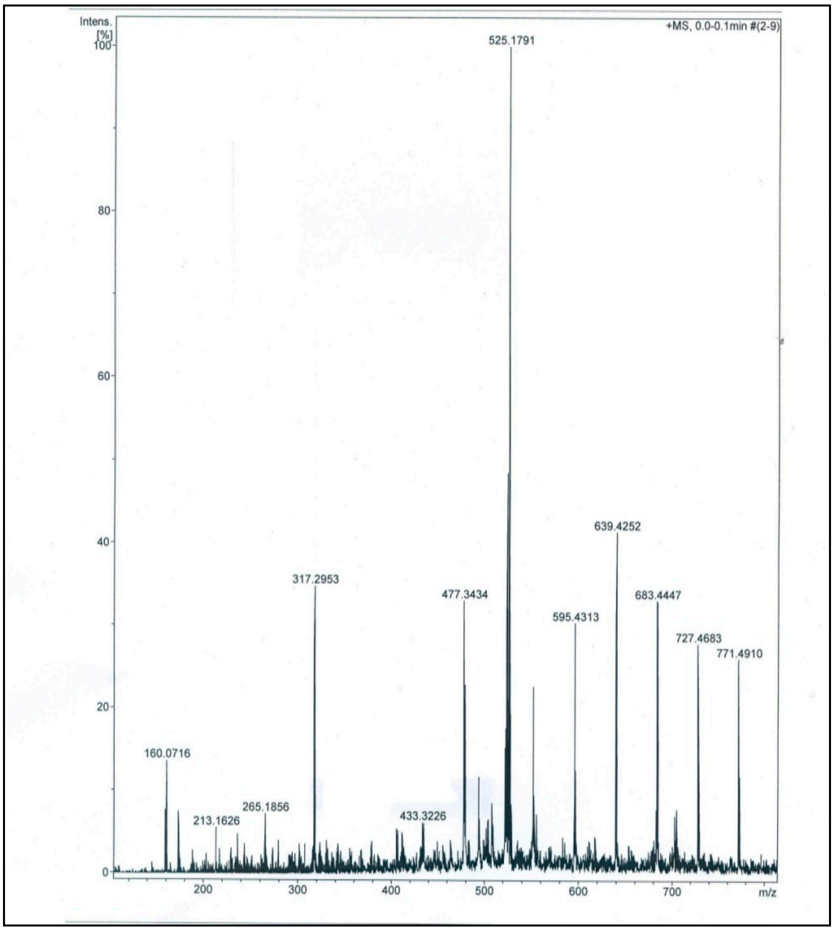

Figure S2. ESI-Mass spectrum of compound **16**. ESI-MS (*m*/*z*) Calcd, $C_{26}H_{22}N_4O_2Se$: 502.4, found: 525.1 $[M + Na]^+$.

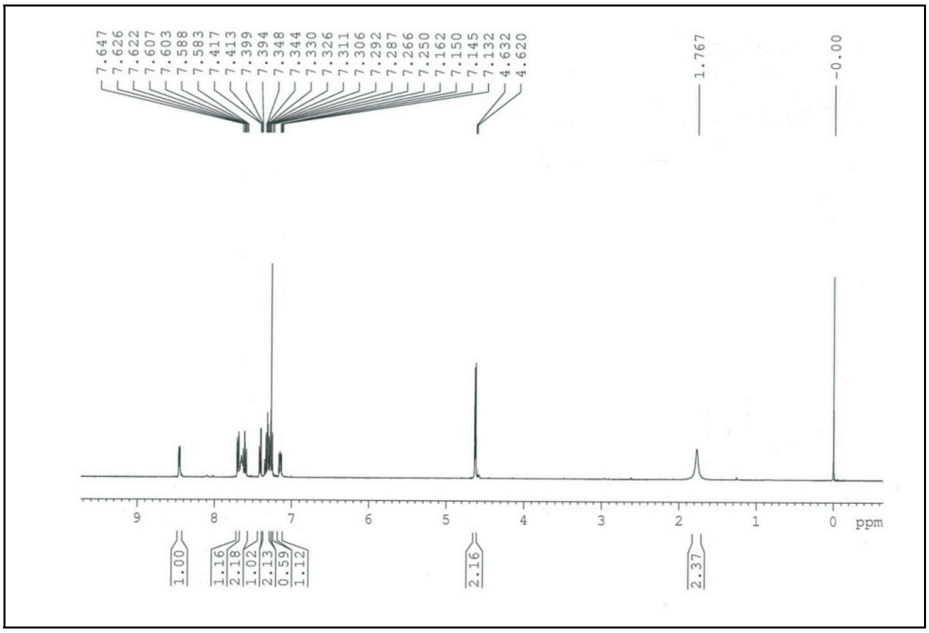

Figure S3. ^{1}H-NMR spectra of pure spirodiazaselenurane **30** in $CDCl_3$.

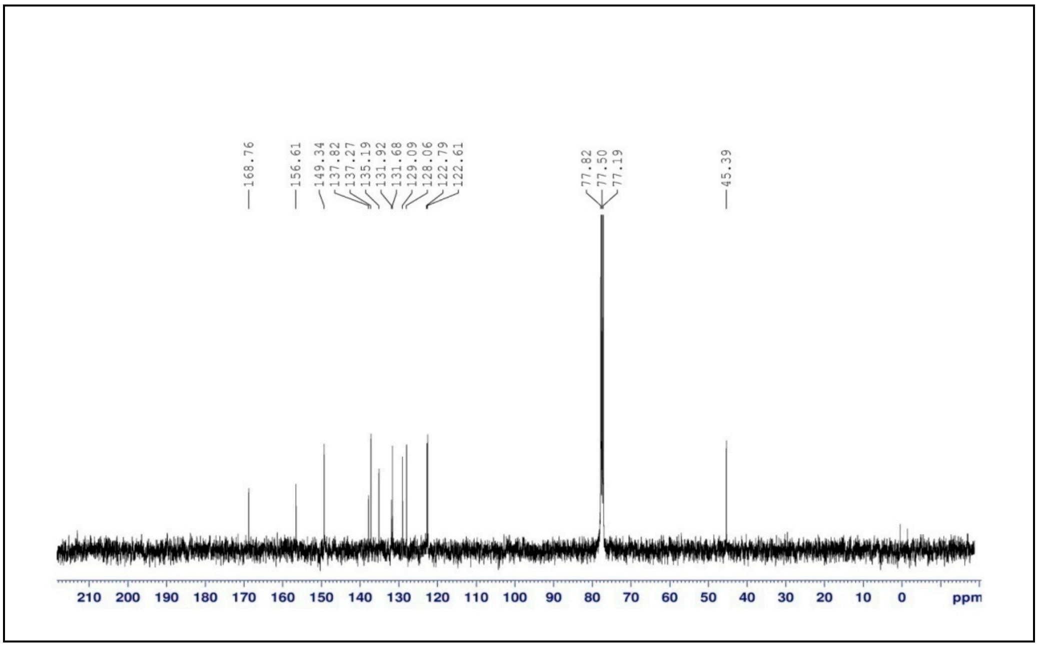

Figure S4. ^{13}C-NMR spectra of pure spirodiazaselenurane **30** in DMSO-d_6.

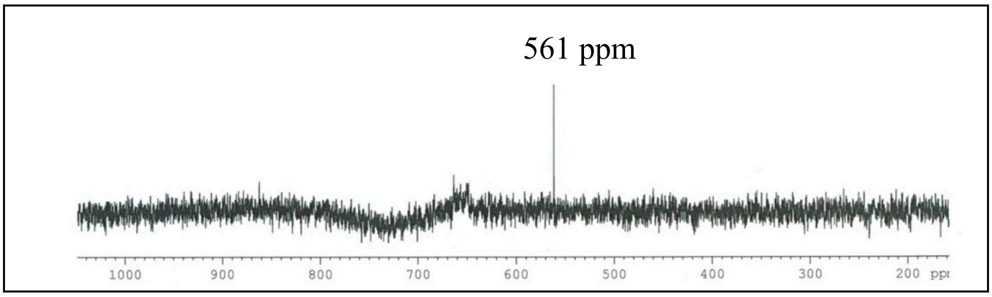

Figure S5. ^{77}Se-NMR spectra of pure spirodiazaselenurane **30** in DMSO-d_6.

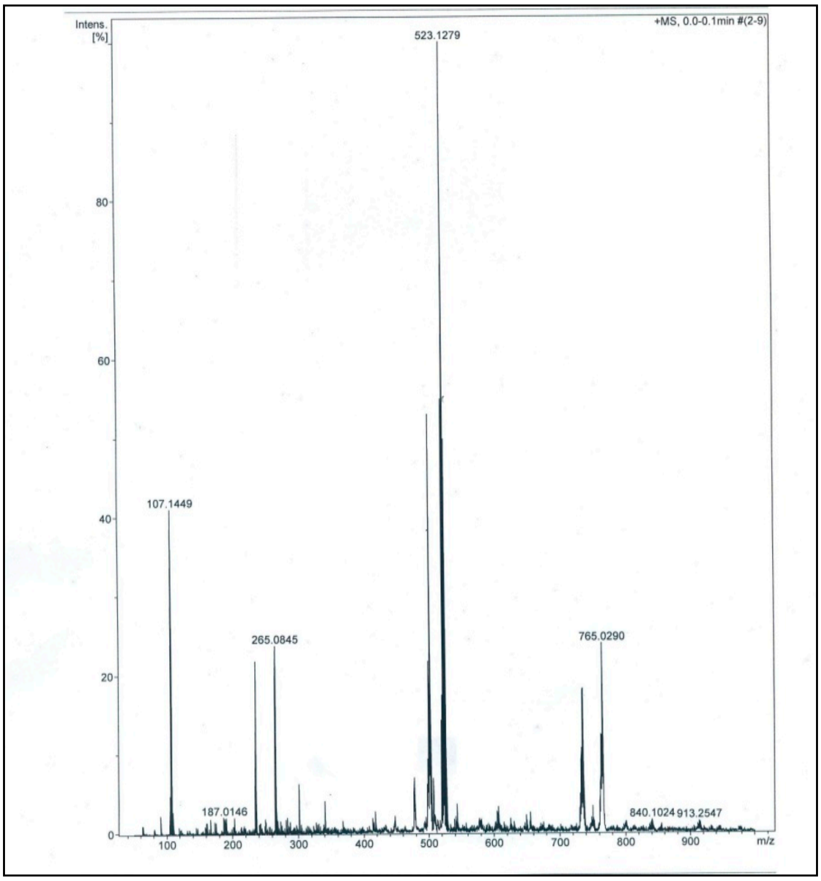

Figure S6. ESI-Mass spectrum of compound **30**. ESI-MS (*m*/*z*) Calcd, $C_{26}H_{20}N_2O_4Se$: 503.4, found: 523.1 $[M + Na]^+$.

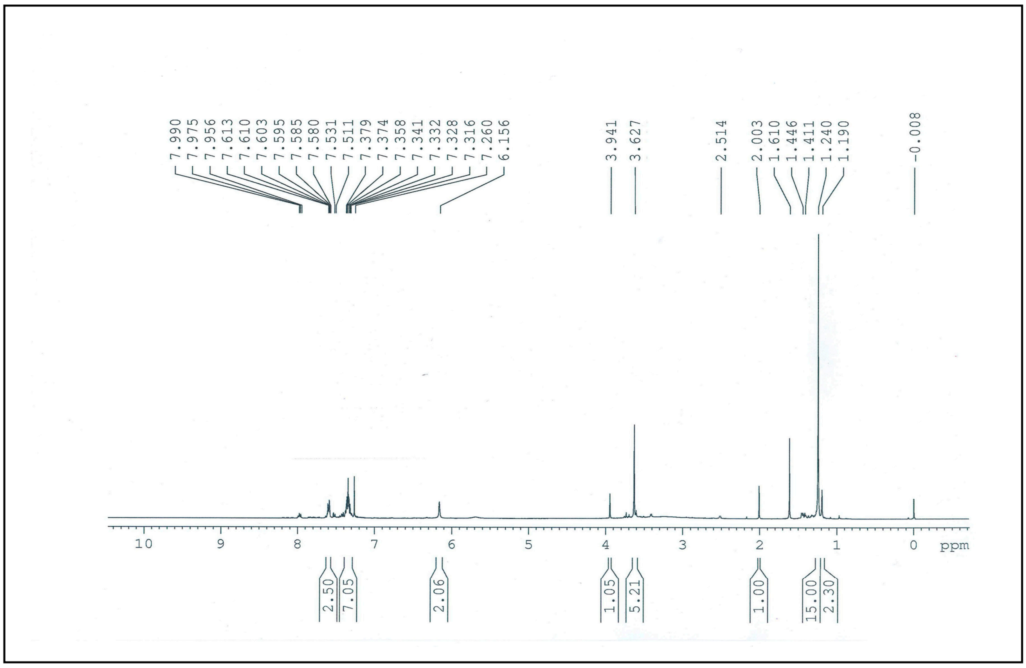

Figure S7. ^{1}H-NMR spectra of pure spirodiazaselenurane **34** in $CDCl_3$.

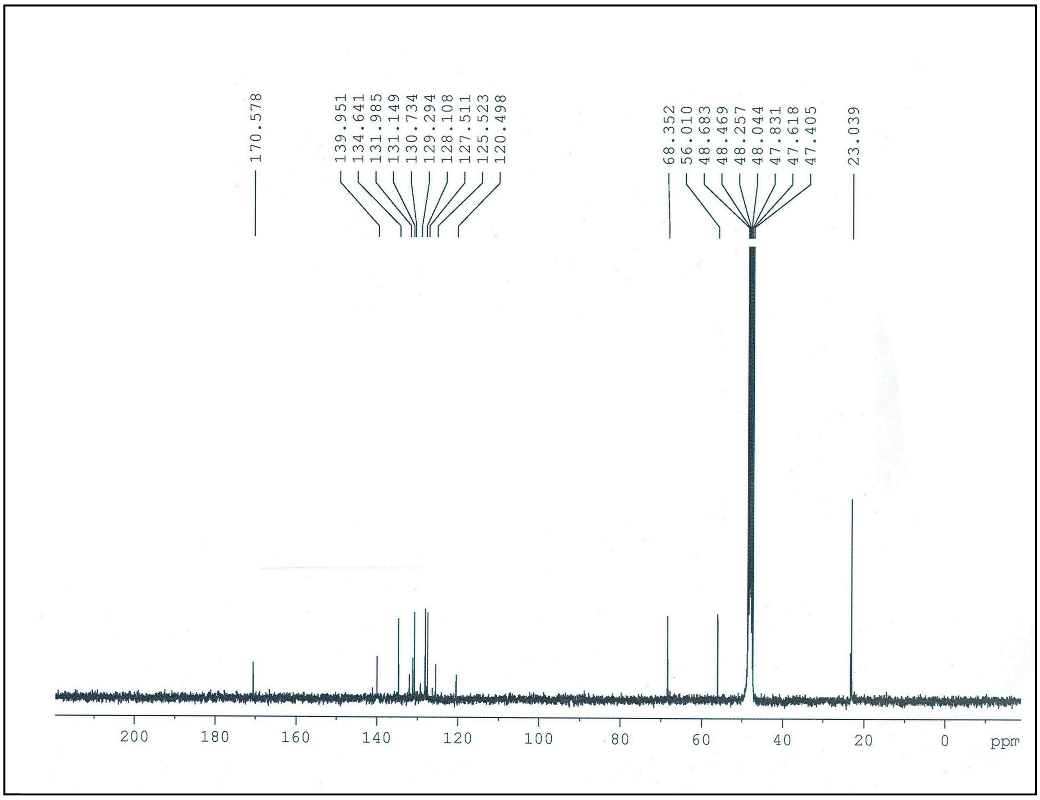

Figure S8. ^{13}C-NMR spectra of pure spirodiazaselenurane **34** in $CDCl_3$.

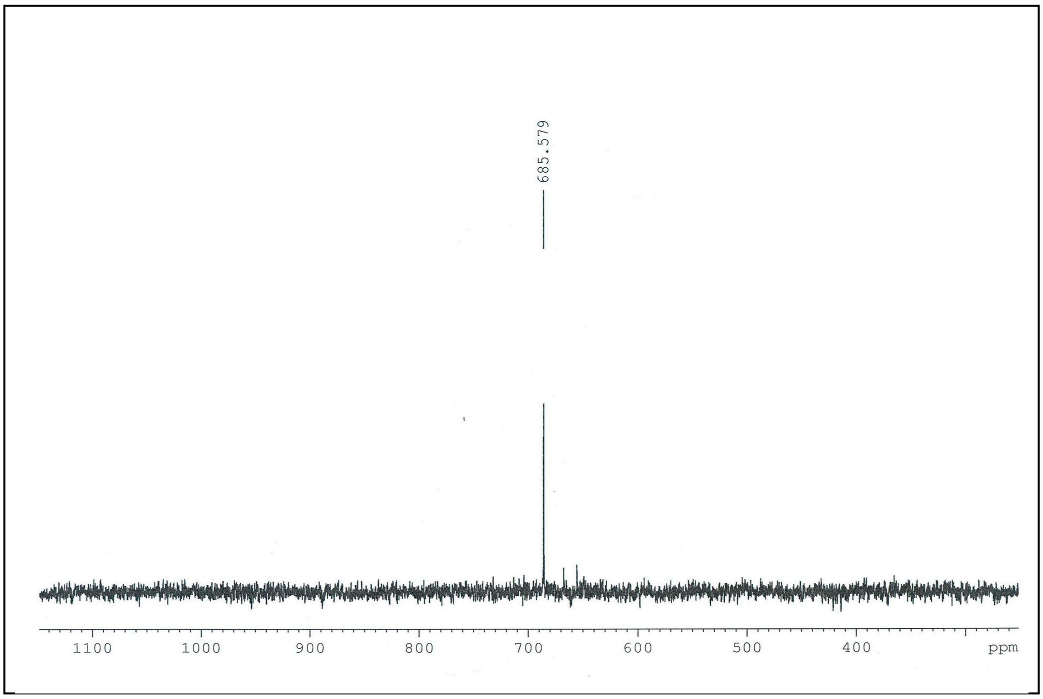

Figure S9. ^{77}Se-NMR spectra of pure spirodiazaselenurane **34** in $CDCl_3$.

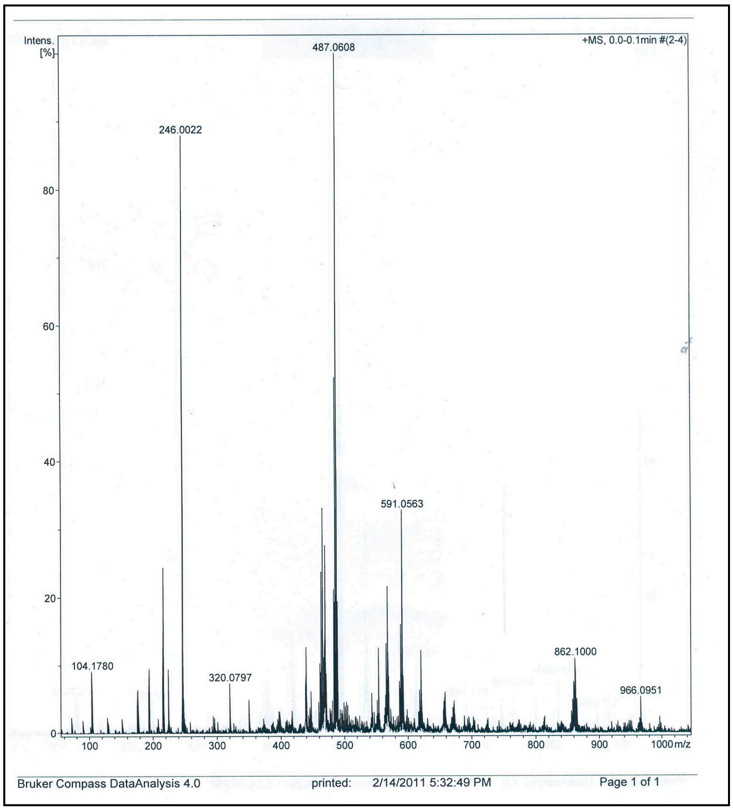

Figure S10. ESI-Mass spectrum of compound **34**. ESI-MS, (*m*/*z*) Calcd, $C_{22}H_{26}N_2O_4Se$: 462.4, found: 487.0 $[M + Na]^+$.

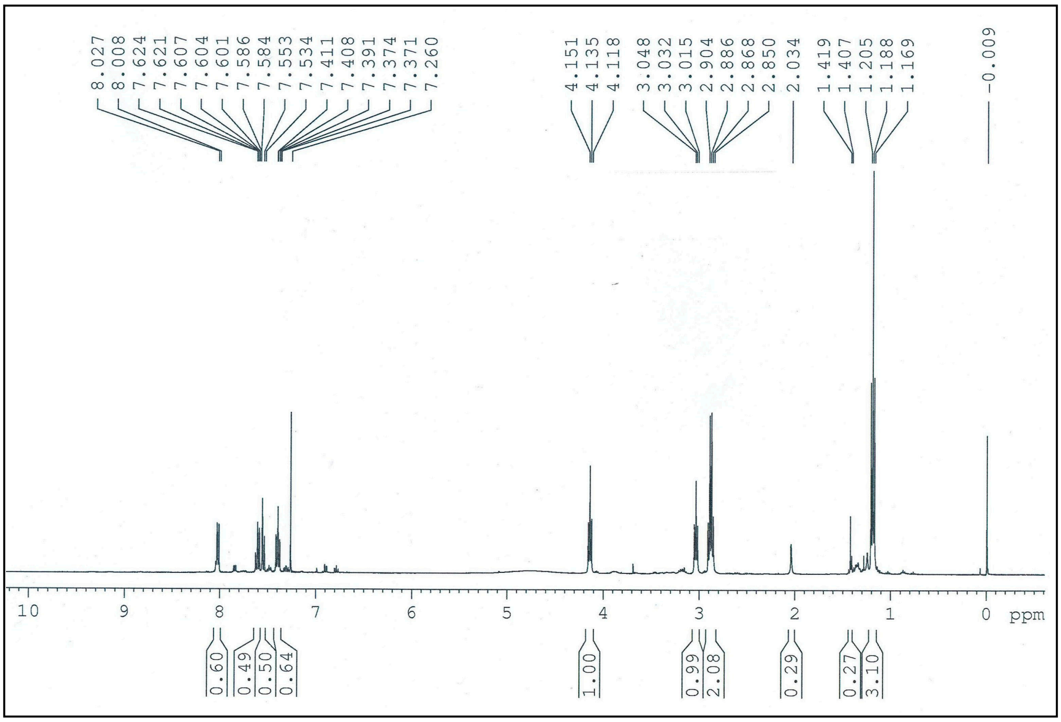

Figure S11. ^{1}H-NMR spectra of pure spirodiazaselenurane **35** in $CDCl_3$.

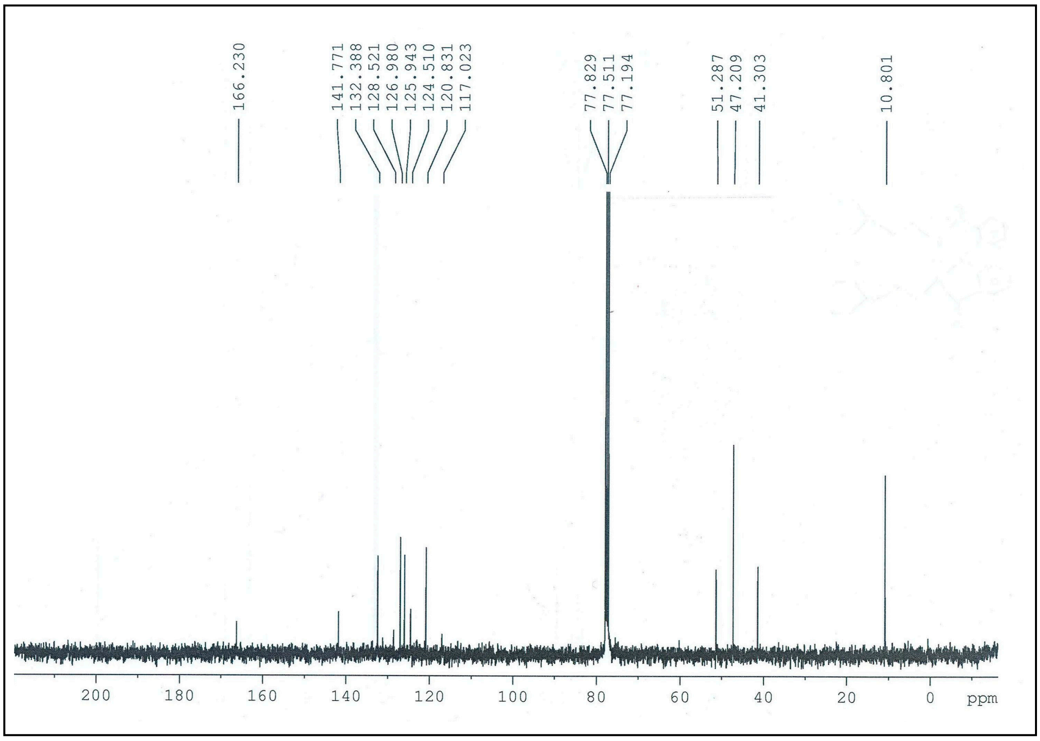

Figure S12. ^{13}C-NMR spectra of pure spirodiazaselenurane **35** in $CDCl_3$.

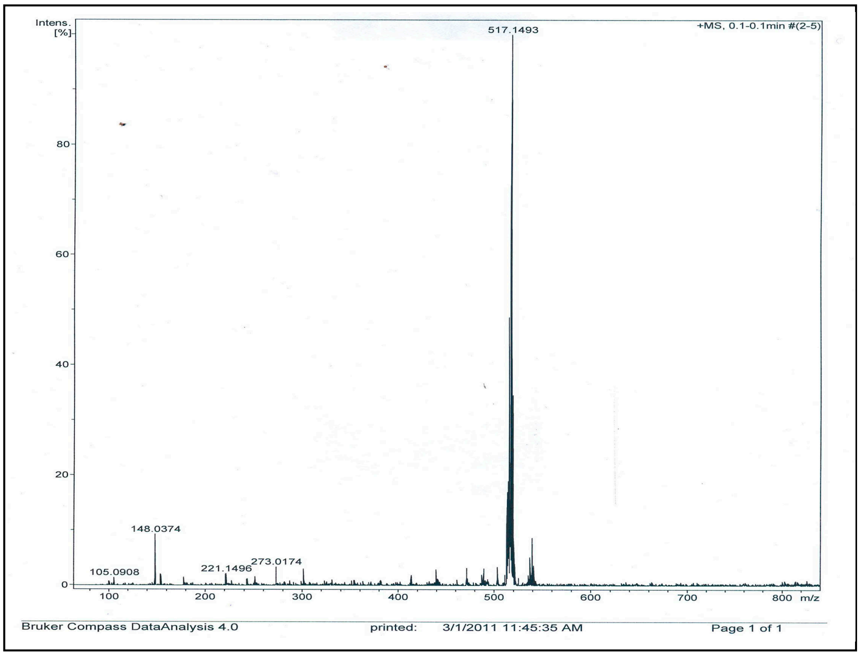

Figure S13. ESI-Mass spectrum of compound **35**. Calcd, $C_{26}H_{36}N4O_2Se$: 516.0, found: 517.1 $[M + H]^+$.

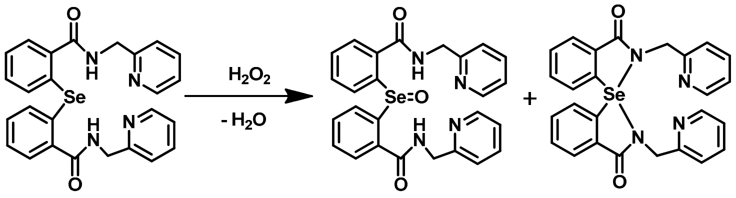

16 23 30

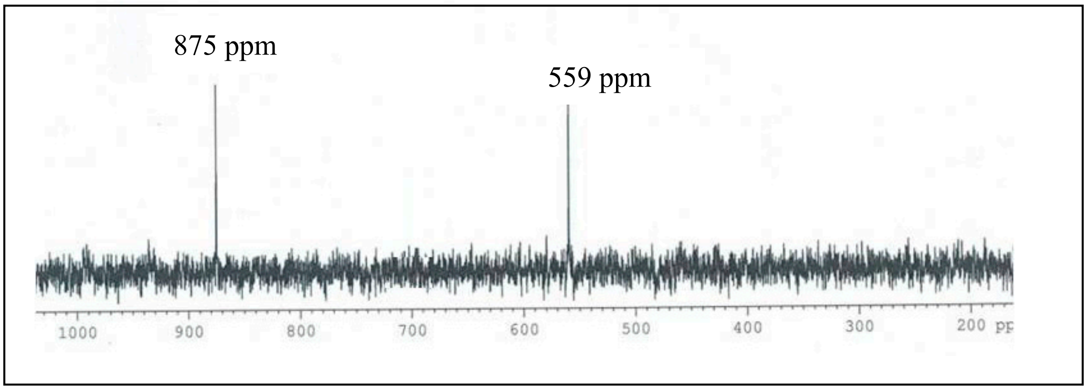

Figure S14. ^{77}Se-NMR spectrum of the diaryl selenide **16** with 1 equiv of H_2O_2 in CH_2Cl_2. Formation of selenoxide **23** and spirodiazaselenurane **30** were observed.

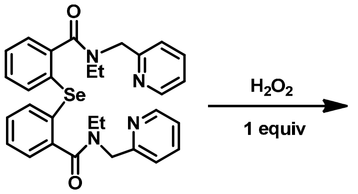

36 37

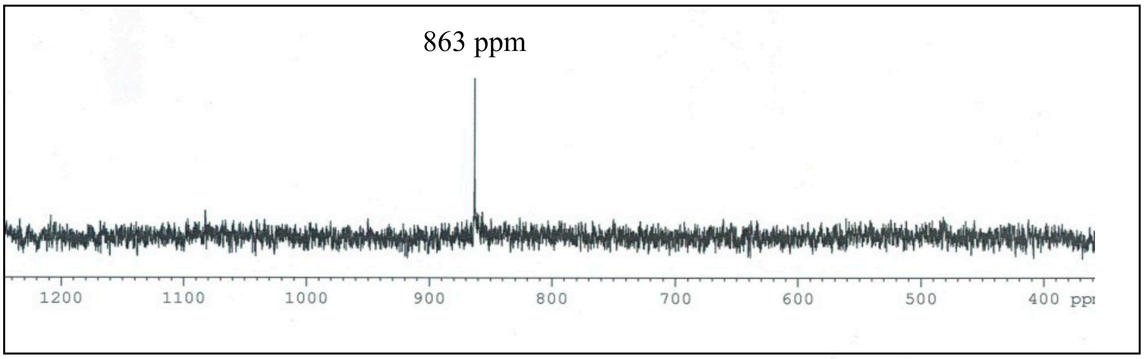

Figure S15. ^{77}Se-NMR spectrum of the monoselenide **36** with 1 equiv of H_2O_2 in CH_2Cl_2. Formation of the selenoxide **37** was observed.

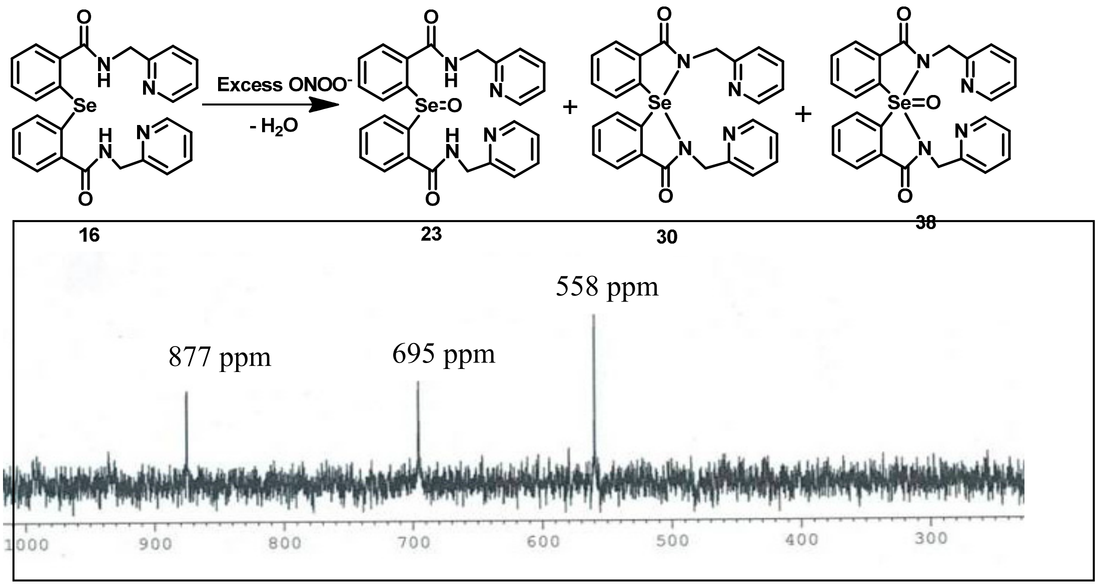

Figure S16. ^{77}Se-NMR spectrum of the diaryl selenide **16** with excess of $ONOO^-$ in CH_2Cl_2. Formation of selenoxide **23**, spirodiazaselenurane **30** and selenurane oxide **38** were observed.

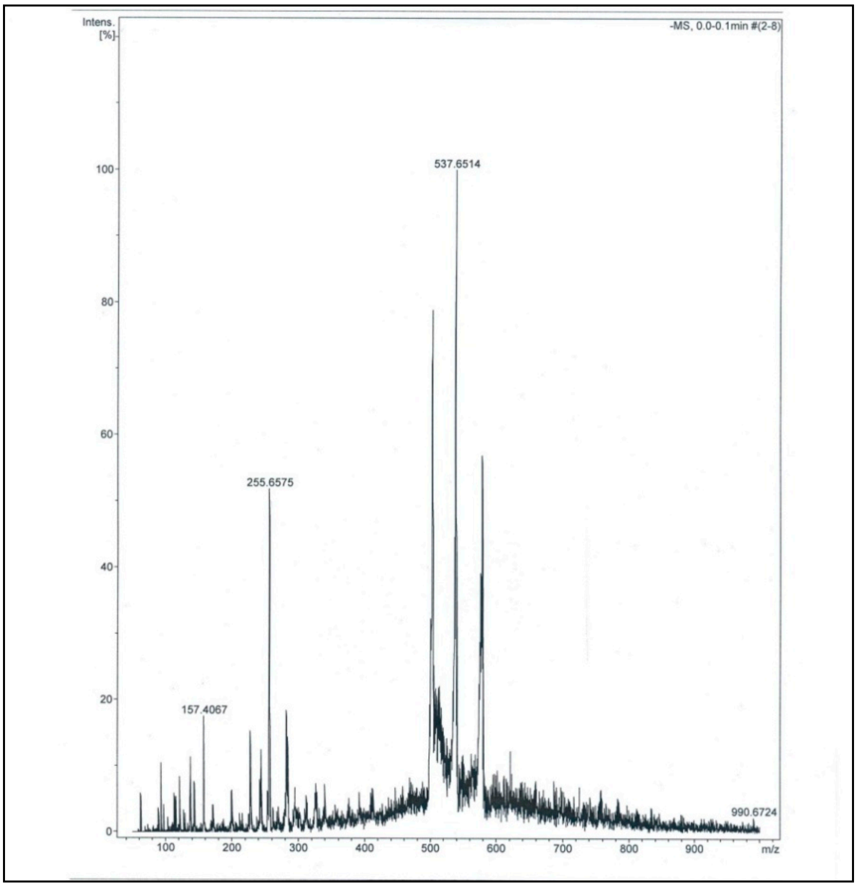

Figure S17. ESI-Mass spectrum of diaryl selenide **16** upon addition of excess of $ONOO^-$ in CD_3OD. Calculated Mass for compound **30**: 500.07, Observed Mass: 501.91 $[M + H]^+$, Calculated Mass for compound **38**, Calculated Mass: 516.07, Observesd Mass: 537.65 $[M + Na]^+$.

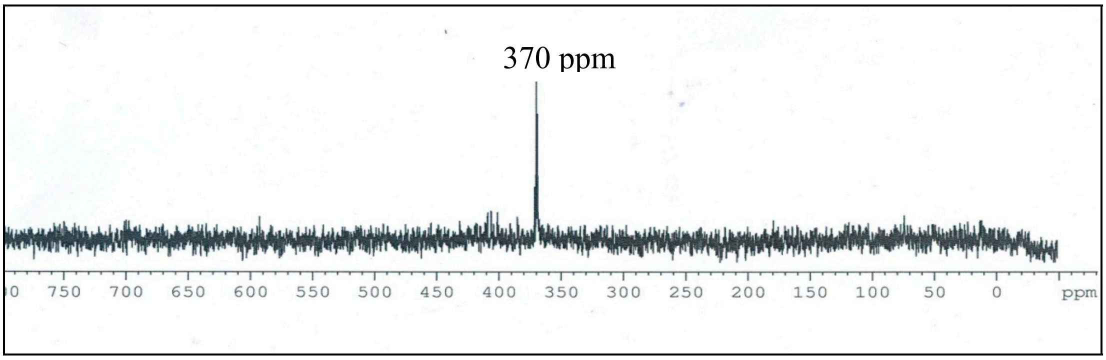

Figure S18. ^{77}Se-NMR spectra of pure monoselenide **36** in $CDCl_3$.

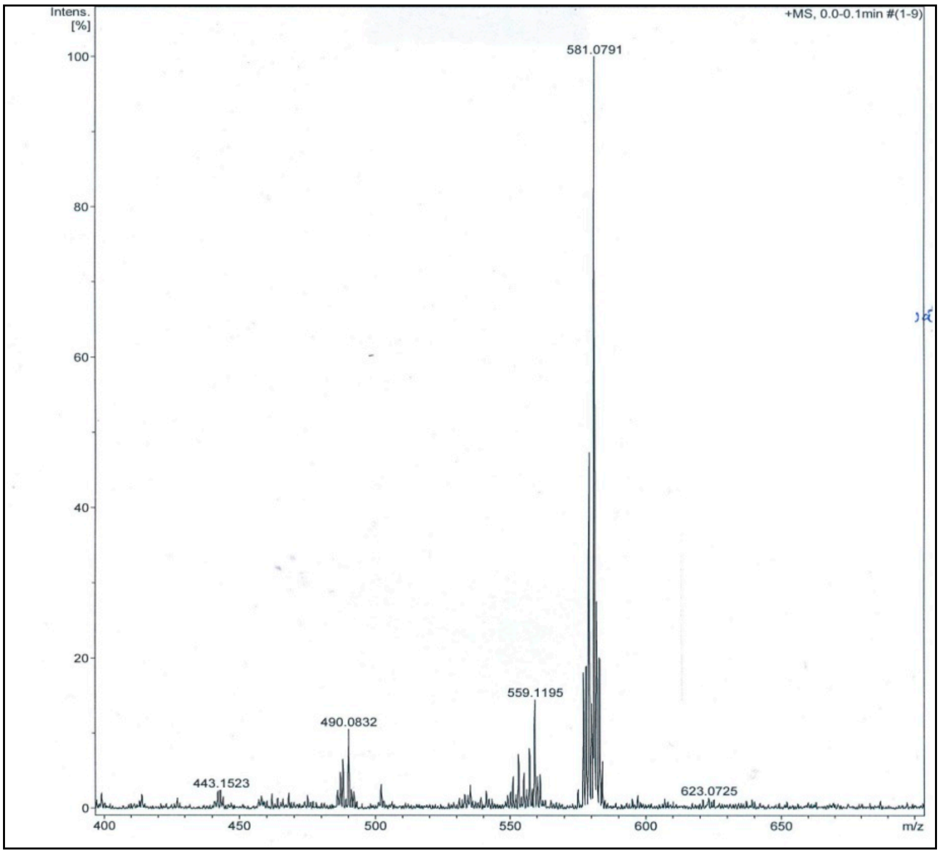

Figure S19. ESI-Mass spectrum of compound 36. ESI-MS (*m*/*z*) Calcd, $C_{30}H_{30}N_4O_2Se$: 558.1, found: 581.0 $[M + Na]^+$.

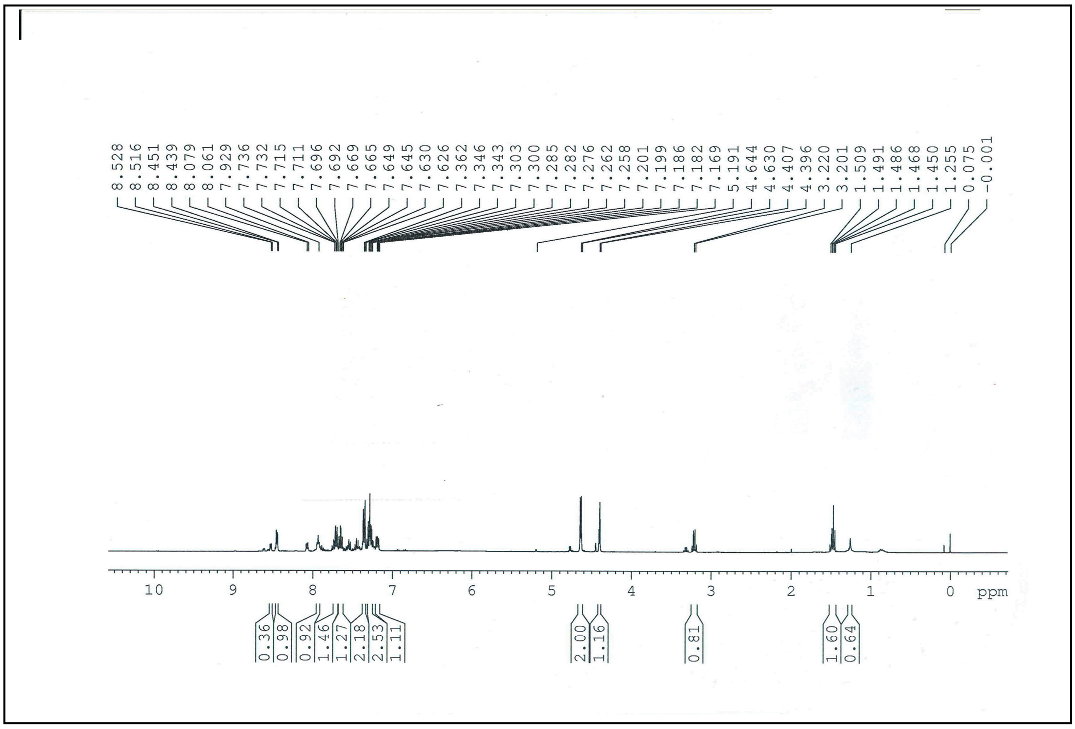

Figure S20. ^{1}H-NMR spectra of pure selenoxide **37** in $CDCl_3$.

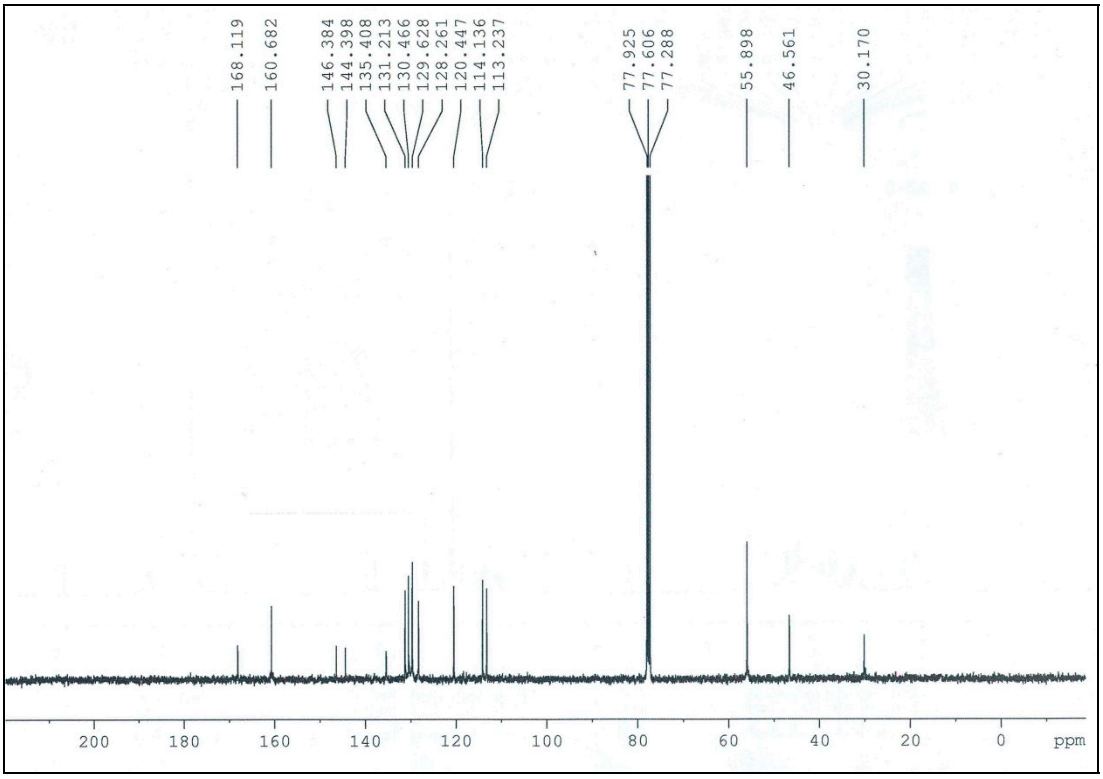

Figure S21. ^{13}C-NMR spectra of pure selenoxide **37** in $CDCl_3$.

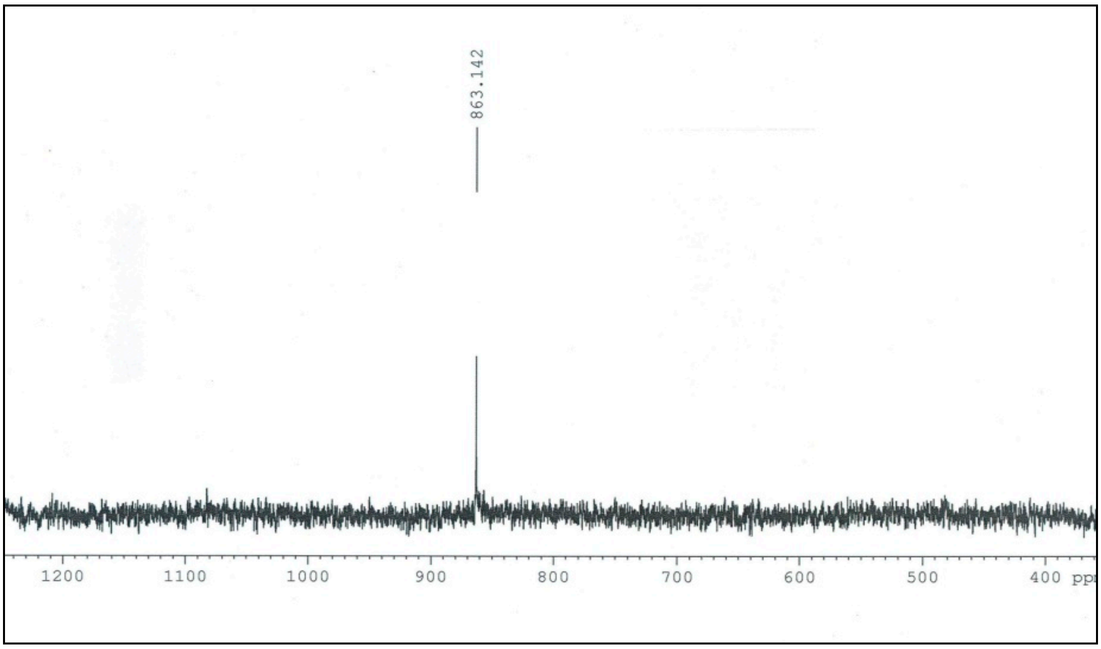

Figure S22. ^{77}Se-NMR spectra of pure selenoxide **37** in $CDCl_3$.

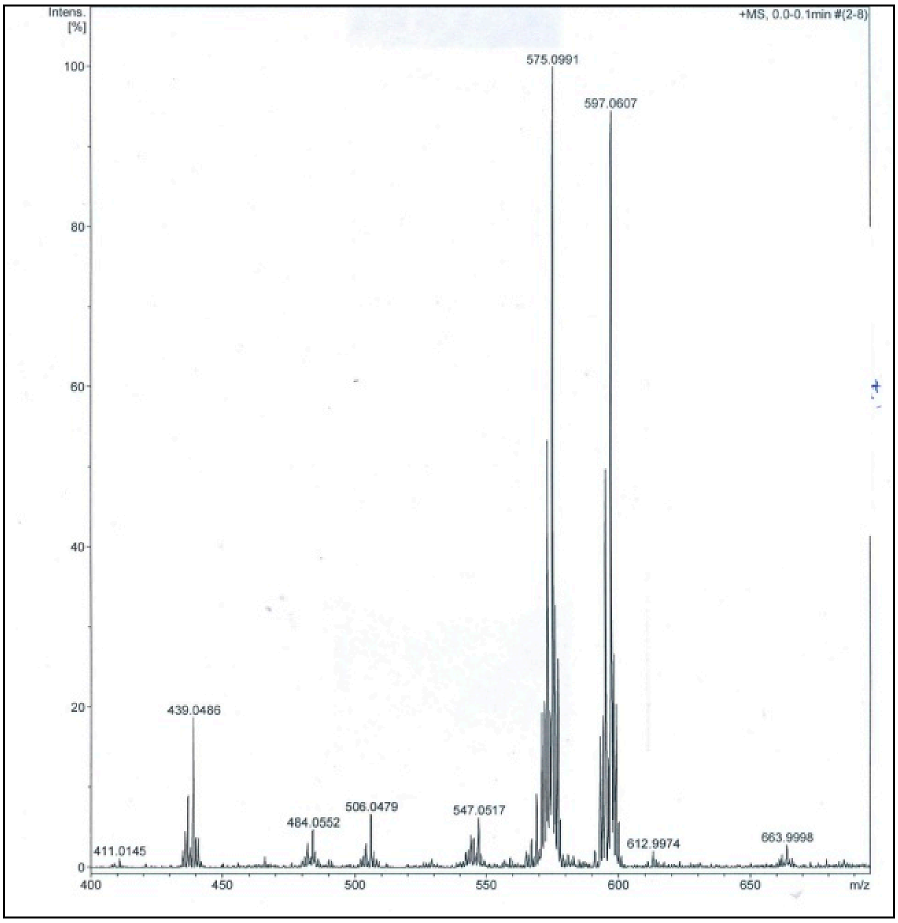

Figure S23. ESI-Mass spectrum of compound 37. ESI-MS (*m*/*z*) Calcd, $C_{30}H_{30}N_4O_3Se$: 574.6, found: 575.1.0 $[M + H]^+$.

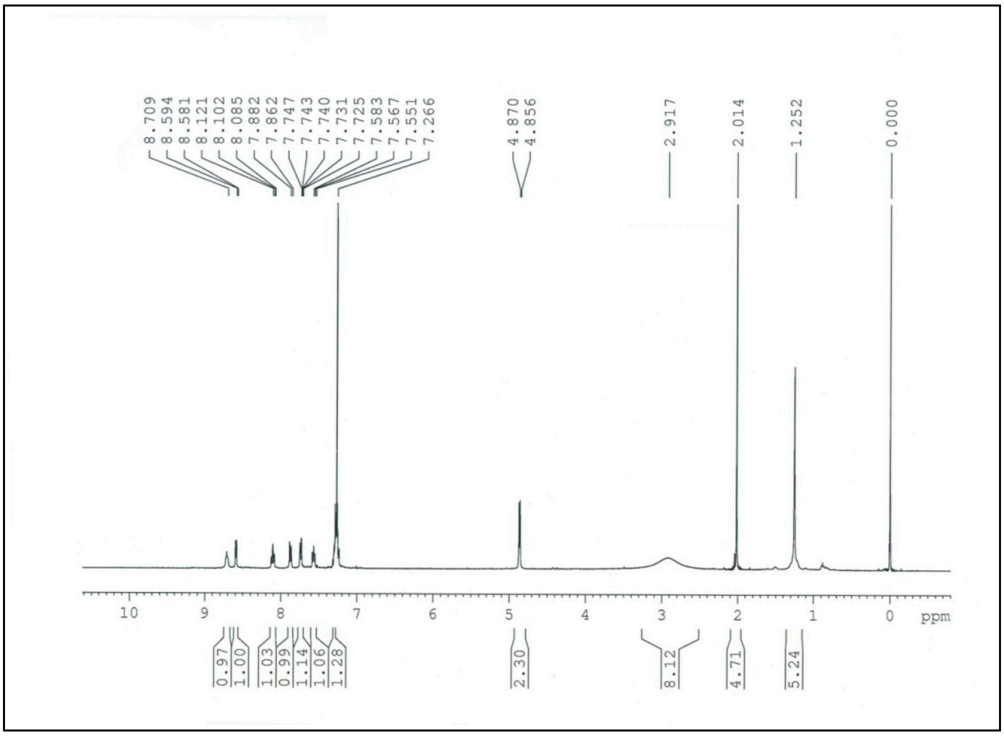

Figure S24. ^{1}H-NMR spectra of pure selenurane oxides **38** in $CDCL_3$.

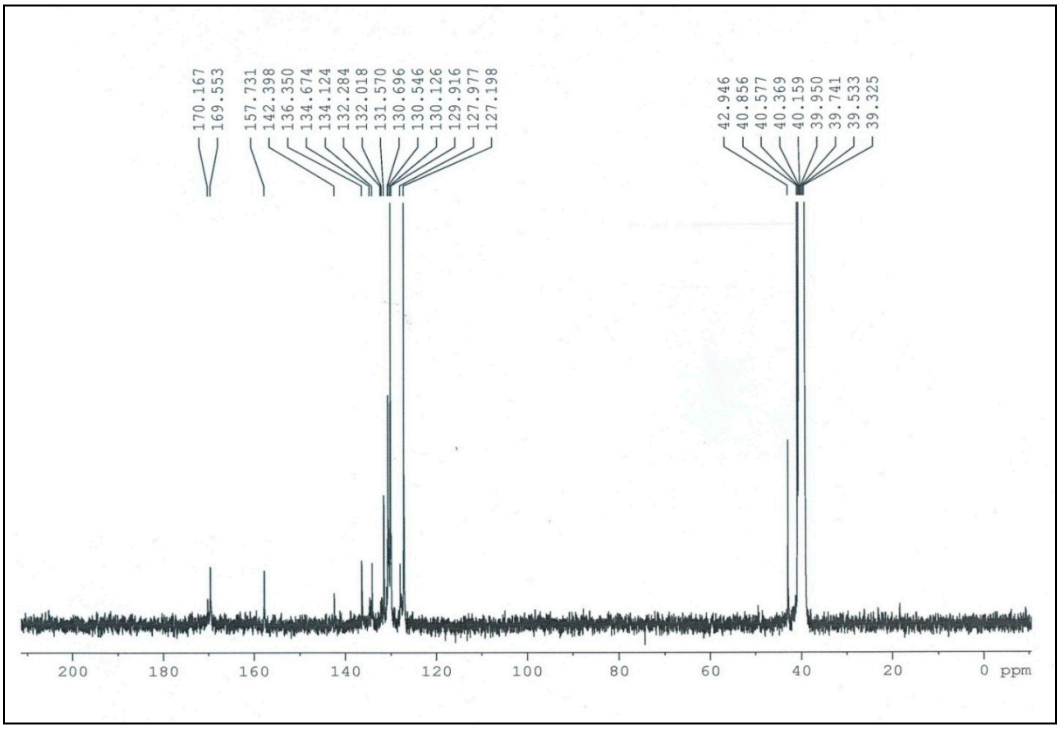

Figure S25. ^{13}C-NMR spectra of pure selenurane oxides **38** in DMSO-d_6.

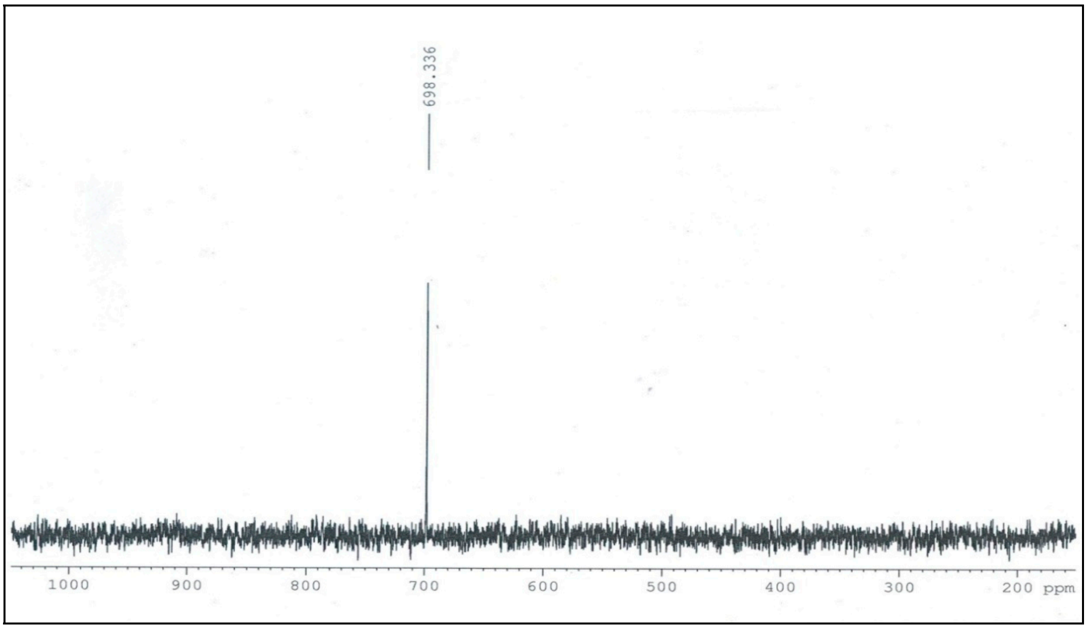

Figure S26. ^{77}Se-NMR spectra of pure spirodiazoselenurane **38** in DMSO-d_6.

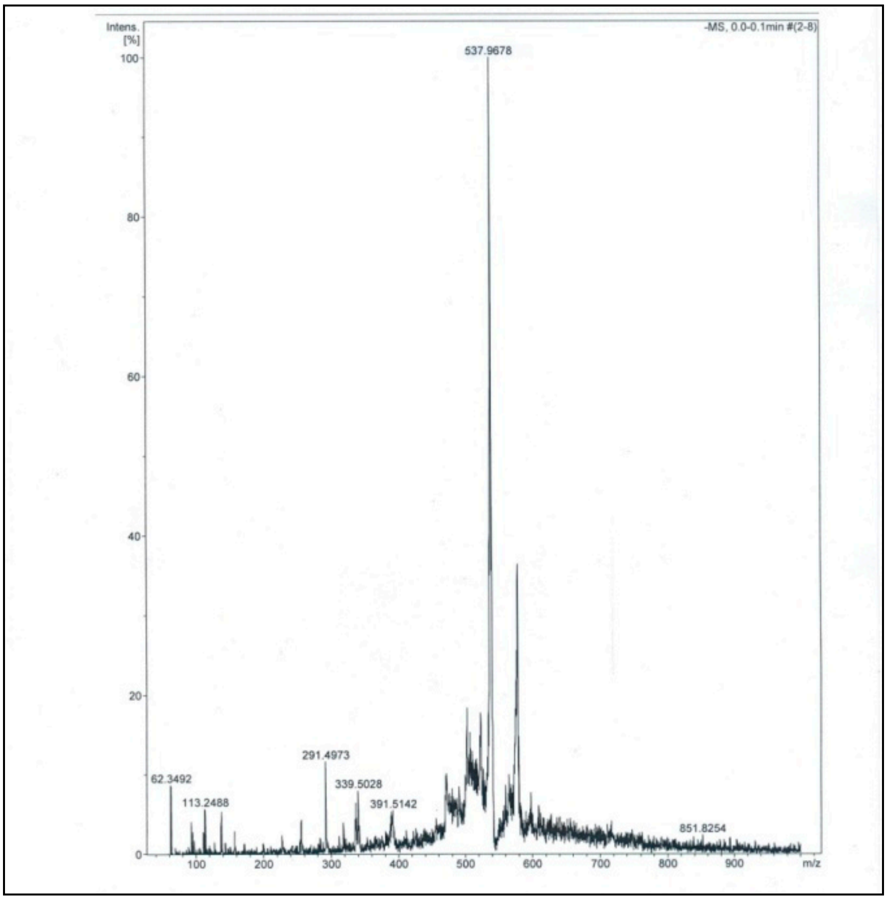

Figure S27. ESI-Mass spectrum of compound **38.** ESI-MS (*m*/*z*) Calcd, $C_{26}H_{20}N4O_3Se$: 516.8, found: 537.9 $[M + Na]^+$.
